# Peroxisomes, PPARs, and Their Role in Macrophages

**DOI:** 10.3390/cells14242021

**Published:** 2025-12-18

**Authors:** Anggi Muhtar Pratama, Heike Bömmel, Hevi Wihadmadyatami, Yudy Tjahjono, Süleyman Ergün, Akash Bachhuka, Srikanth Karnati

**Affiliations:** 1 Institute of Anatomy and Cell Biology, Julius-Maximilians-Universität Würzburg, 97070 Würzburg, Germany or anggi.muhtar.p@mail.ugm.ac.id (A.M.P.); heike.boemmel@uni-wuerzburg.de (H.B.); or suleyman.ergun@atlas.edu.tr (S.E.); 2 Department of Pharmacology, Faculty of Veterinary Medicine, Universitas Gadjah Mada, Yogyakarta 55281, Indonesia; 3 Department of Anatomy, Faculty of Veterinary Medicine, Universitas Gadjah Mada, Yogyakarta 55281, Indonesia; heviwihadmadyatami@ugm.ac.id; 4 Department of Biomedical Science, Faculty of Pharmacy, Universitas Katolik Widya Mandala Surabaya, Surabaya 60237, Indonesia; yudy.tjahjono@ukwms.ac.id; 5 Atlas University Research Center (ARC), Atlas University, No. 40, Istanbul 34403, Turkey; 6 Institute of Chemical Research of Catalonia (ICIQ), The Barcelona Institute of Science and Technology, Av. Països Catalans 16, 43007 Tarragona, Spain; 7 Comprehensive Heart Failure Center, University Clinic Würzburg, 97078 Würzburg, Germany

**Keywords:** peroxisomes, peroxisome proliferator-activated receptors (PPARs), macrophages, immune cells, macrophage polarization, M1 phenotype, M2 phenotype, inflammation, oxidative stress, lipid metabolism

## Abstract

Macrophages are versatile immune cells capable of modifying their functions based on their location and the specific requirements of the immune response. They polarize into the M1 phenotype when stimulated by inflammatory agents. In contrast to resolve inflammation and to facilitate tissue repair, macrophages polarize into the M2 phenotype. Polarization alters the cellular composition of the macrophages, including peroxisomes and peroxisome proliferator-activated receptors (PPARs). In macrophages, peroxisomes and PPARs perform at least three key roles: mediating inflammation, reducing oxidative stress, and regulating lipid metabolism. We review the functional role of peroxisomes and PPARs on macrophage biology focusing on adaptive mechanisms during these processes. The insights gained from this analysis are expected to lead to new advancements in treating inflammation and immune-related disorders, including autoimmune disorders, metabolic inflammation, and neurodegenerative conditions.

## 1. Introduction

During embryonic development, macrophages are derived from erythro-myeloid progenitors (EMPs). In adulthood, they are produced in the bone marrow, circulate through the bloodstream, and subsequently differentiate within various organs [1]. Once they reach their target organs, resident macrophages are classified based on their anatomical location, for instance, they are referred to as Kupffer cells in the liver and Microglia in the central nervous system (CNS) [2]. Macrophages are essential components of the innate immune system, play a crucial role in regulating inflammation, and cellular metabolism [1,3,4]. In response to inflammatory conditions, macrophages polarize into the classically activated macrophage (M1) phenotype. These cells sustain inflammation by releasing pro-inflammatory cytokines and recruiting other immune cells, such as T lymphocytes. To resolve inflammation and promote tissue repair, macrophages transition to the alternatively activated macrophage (M2) phenotype, which releases anti-inflammatory mediators and facilitates wound healing [5,6].

In carrying out these specific functions, macrophages utilize organelles like peroxisomes. Peroxisomes are abundant in microglia [7,8], alveolar macrophages, peritoneal macrophages, bone marrow-derived macrophages (BMDMs), and the RAW264.7 macrophage-like cell line [9,10]. Peroxisomes are multifunctional single-membrane organelles essential for regulating inflammation, lipid synthesis, and maintaining oxidative balance. They break down inflammatory substances, such as prostaglandins and leukotrienes, and produce anti-inflammatory mediators like docosahexaenoic acid (DHA) and eicosapentaenoic acid (EPA) [9,10,11,12,13]. Further, peroxisomes play a crucial role in plasmalogen, cholesterol, and bile acid synthesis, supporting inflammatory processes [6,14]. They produce antioxidant enzymes that help eliminate reactive oxygen species (ROS) and reactive nitrogen species (RNS), thereby maintaining redox balance and reducing oxidative stress [10]. These organelles adapt to morphological and functional changes within a cell in response to environmental stimuli [15,16]. Their activity is tightly regulated by PPARs, key transcription factors that govern peroxisomal gene expression [9,12].

The loss of peroxisomes leads to lipid accumulation, particularly very long-chain fatty acids (VLFCAs), increasing ROS and RNS production, and impairing lipid synthesis. This can disrupt mitochondrial and endoplasmic reticulum (ER) activity, and trigger inflammatory signaling through nuclear factor-kappa B (NF-κB) activation [13]. Therefore, disturbances or damages to peroxisomes can lead to various serious diseases, such as X-linked adrenoleukodystrophy (X-ALD), Zellweger syndrome (ZS), Heimler syndrome (HS), neonatal adrenoleukodystrophy (NALD), infantile refsum disease (IRD), and rhizomelic chondrodysplasia punctata (RCDP) [13,17,18,19]. Bachhuka et al., 2022 [11] further added that degenerative diseases such as autoimmune diseases, diabetes, and stroke may manifest when there is damage or deficiency in peroxisomes.

Moreover, He et al., 2021 [14] reported that peroxisomes play a crucial role in cellular metabolism through dynamic interactions with other organelles, including mitochondria, ER, ribosomes, and lysosomes. Mitochondria share proteins for peroxisomal biogenesis and cooperate with peroxisomes in fatty acid oxidation. Moreover, the ER supports peroxisome function by supplying key proteins required for biogenesis and collaborating in the synthesis of plasmalogens and cholesterol. Free ribosomes contribute to matrix proteins required for peroxisomal biogenesis, while lysosomes assist in cholesterol synthesis in conjunction with peroxisomes [20]. PPARs, which regulate peroxisomal gene transcription, play a critical role in regulating cell differentiation, development, and metabolism. This includes the regulation of carbohydrate, lipid, and protein metabolism. Disturbance in these metabolic pathways results in tumorigenesis and other diseases [21,22,23].

Given their pivotal role in both immune regulation and metabolism, elucidating the functions of peroxisomes and PPARs in macrophages is critical for understanding how metabolic dysfunction intersects with immune responses. This review explores their involvement in inflammation, redox control, ferroptosis, efferocytosis, and macrophage polarization, highlighting the implications of peroxisomal dysfunction and its contribution to the pathogenesis of many diseases. To comprehensively address these challenges, it is important to examine the roles of peroxisomes and PPARs in macrophages. As a prerequisite, a clear understanding of peroxisomal biogenesis, protein import mechanisms, and core metabolic functions of peroxisomes and PPARs is necessary.

## 2. Development of Peroxisomes

**Figure 1:**
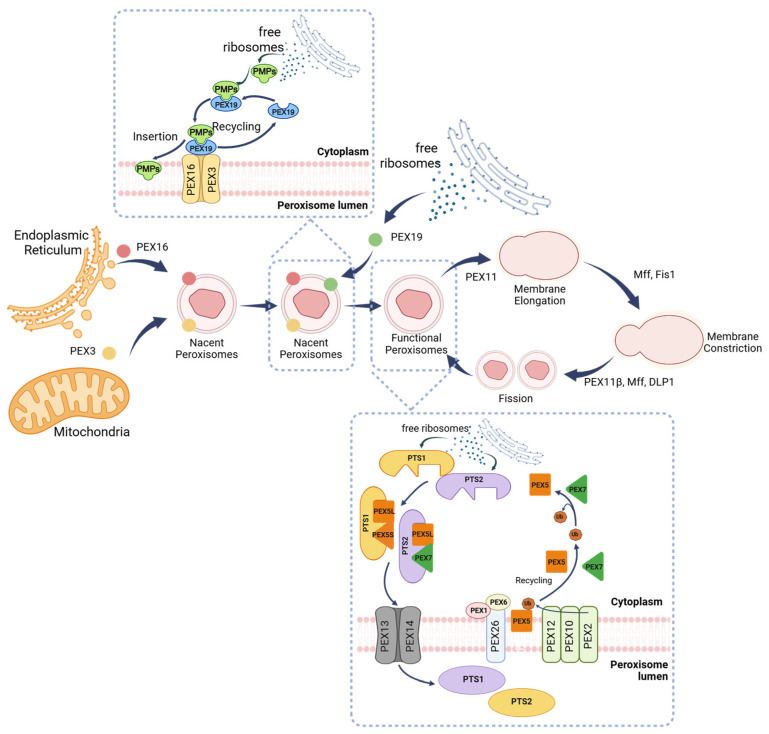
Process of Peroxisome Biogenesis. There are three important stages that occur during the biogenesis of peroxisomes: the formation of the lipid membrane, the import of matrix proteins, and the subsequent enlargement of peroxisomes. The formation of the peroxisomal membrane begins with the fusion of pre-peroxisomal vesicles that originate from the mitochondria and the endoplasmic reticulum. Once budding takes place, peroxisomal membrane proteins are transported from free ribosomes. Matrix proteins synthesized on free ribosomes contain either peroxisomal targeting signals 1 or 2. Once these matrix proteins dock at the PEX13/PEX14 complex site, the peroxisomal targeting signals 1 and 2 are released. The process of peroxisome enlargement involves elongation, constriction, and fission. The resulting two asymmetric daughter peroxisomes then mature and become functional by importing additional matrix and membrane proteins. This process may lead to a re-entry into the membrane expansion phase of the cycle. Abbreviations: PEX (Peroxin), PMPs (Peroxisomal membrane proteins), PTS (Peroxisomal targeting signals), Mff (Mitochondrial fission factor), DLP1 (Dynamin-like protein 1), Fis1 (Mitochondrial fission protein 1). Created in BioRender. Wihadmadyatami, H. (2025) https://BioRender.com/dg41se9 (accessed on 10 December 2025).

Peroxisome development involves three essential processes: formation of lipid membrane, import of matrix proteins, and subsequent enlargement of the organelle (Figure 1) [24,25]. Peroxisomal membrane formation is initiated by the fusion of pre-peroxisomal vesicles originating from the mitochondria (PEX3 and PEX14) and the ER (PEX16). Once budding occurs, peroxisomal membrane proteins (PMPs) are transported by PEX19 from free ribosomes and anchored onto PEX3 [11,14,25,26,27].

Matrix proteins produced on free ribosomes contain peroxisomal targeting signals at either the C terminus (PTS1) or the N terminus (PTS2) [14,28]. PTS1 proteins are delivered to peroxisomes by homo-oligomers and hetero-oligomers of PEX5S (short) and PEX5L (long), while PTS2 proteins are transported to peroxisomes by PEX5L-PEX7 [17,29,30]. Peroxisomal matrix proteins are imported into the lumen via the PTS1 and PTS2 pathways. The C-terminal PTS1 motif remains in the mature protein, whereas only PTS2-containing proteins undergo N-terminal cleavage upon import, resulting in removal of the PTS2 signal [17]. Generally, both PEX5 and PEX7 are recycled back to the cytosol through a process reliant on an exporter complex consisting of PEX1, PEX6, and PEX26 [14]. Mainly, PEX5 recycling necessitates cysteine mono-ubiquitination, which is mediated by the RING-type ubiquitin ligases PEX2, PEX10, and PEX12 [11,14,26,31].

Following formation of pre-peroxisomes, PEX11 proteins (including PEX11α, PEX11β, and PEX11γ) are crucial for elongation, constriction, and fission of peroxisomes. Mitochondrial fission factor (Mff) and mitochondrial fission protein 1 (Fis1) are positioned at the constrictions of elongated peroxisomal membranes, where Mff recruits dynamin-like protein 1 (DLP1) or dynamin-1-like protein (DNM1L). This process results in a complex comprising PEX11β, Mff, and DLP1, facilitating Mff-mediated fission during peroxisomal division. The resulting two asymmetric daughter peroxisomes mature and become functional by importing further matrix and membrane proteins, potentially re-entering the membrane expansion phase of the cycle [11,17,32]. The presence of DHA also plays a role in triggering peroxisomal elongation and fission [33].

Loss of function or mutations in PEX3, PEX16, or PEX19 lead to the total absence of peroxisomes, resulting in peroxisomal biogenesis disorders within the Zellweger spectrum [14]. These disorders are severe conditions marked by significant liver dysfunction, developmental delays, neurological abnormalities, and early mortality, typically occurring before the age of 2 [14,17]. Interestingly, when PEX5 is inactivated, Catalase, an important antioxidant enzyme in peroxisomes, is redistributed to the cytoplasm, leading to increased resistance against oxidative stress induced by external H_2_O_2_ treatment. Inactivation of PEX5 disrupts the PEX5–PEX14 complex, preventing proper interaction with Catalase and thereby retaining Catalase in the cytosol, where it is crucial for mitigating oxidative stress. Additionally, PEX5 shows reduced efficiency in importing Catalase during acute oxidative stress [14,17,26]. Kim & Bai, 2022 [33] emphasized that genetic defects in PEX11β reduce peroxisomal abundance because PEX11β is essential for peroxisomal proliferation.

## 3. Peroxisomes Are Involved in Lipid Metabolism

Peroxisomes play a role in fatty acid oxidation (FAO) and the synthesis of cholesterol, bile acid, plasmalogen, and polyunsaturated fatty acid (PUFA).

### 3.1. Transport of Fatty Acids into Peroxisomes

**Figure 2:**
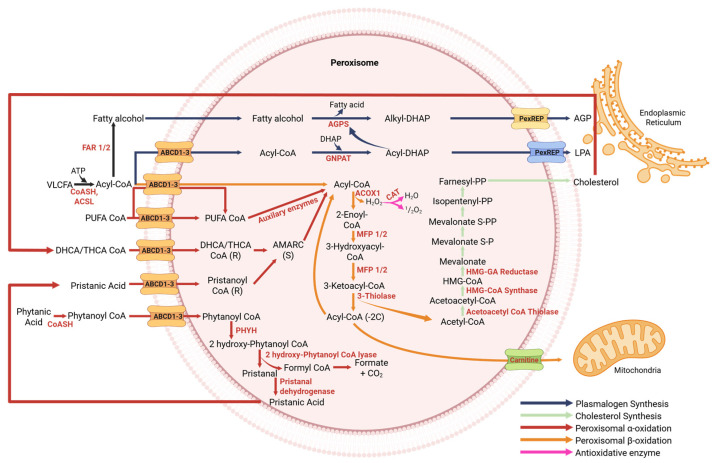
The Role of Peroxisomes. Peroxisomes are essential for fatty acid metabolism, especially in the initial breakdown of VLCFAs and BCFAs through α- and β-oxidation pathways. The shortened fatty acid chains are subsequently transferred to mitochondria for further oxidation and energy production. Additionally, peroxisomes are involved in the biosynthesis of plasmalogens and cholesterol, which are critical components for maintaining cellular membrane integrity and regulating signaling processes. In the regulation of lipid metabolism, peroxisomal ABCD transporters are critical for importing fatty acids into the peroxisome. Moreover, to maintain oxidative balance, peroxisomes generate antioxidative enzymes, such as Catalase, which plays a vital role in scavenging ROS and RNS. Abbreviations: FAR (Fatty acyl-CoA reductase), ABCD (ATP-binding cassette transporter subfamily D), VLCFA (Very Long Chain Fatty Acid), ATP (Adenosine triphosphate), CoASH (Coenzyme A), ACSL (Long-Chain Acyl-CoA Synthetase), PUFA (Polyunsaturated fatty acid), DHCA/THCA (Dihydroxycholestanoic acid/Trihydroxycholestanoic acid), AMARC (α-methylacyl-CoA racemase), PHYH (Phytanoyl-CoA Hydroxylase), AGPS (alkylglycerone phosphate synthase), DHAP (Dihydroxyacetone phosphate), GNPAT (Glyceronephosphate O-Acyltransferase), ACOX (Acyl-coenzyme A oxidase), MFP (MultiFunctional Protein), CAT (Catalase), PexRep (Peroxisomal Reductase Activating PPARγ), AGP (1-alkyl-2-lyso-sn-glycero-3-phosphate), LPA (Lysophosphatidic acid), HMG (Hydroxymethylglutaryl). Created in BioRender. Wihadmadyatami, H. (2025) https://BioRender.com/3vium8c (accessed on 10 December 2025).

Fatty acid transport into peroxisomes is facilitated by the ATP-binding cassette (ABC) transporter subfamily D (ABCD transporter) (Figure 2), such as ABCD1, ABCD2, and ABCD3 [11,14,34]. These proteins import a range of fatty acids, including VLCFAs, long-chain unsaturated fatty acids (LCUFAs), and branched-chain fatty acids (BCFAs) [35].

Fatty acids must be esterified into fatty acyl-CoA by the acyl-CoA synthetase long-chain (ACSL) enzyme before being transported into peroxisomes. Two models of peroxisomal import have been proposed: direct import of fatty acyl-CoAs into the matrix and hydrolysis during translocation is followed by re-esterification within the lumen. PEX19 serves as a chaperone for peroxisomal ABCD transporters, preventing their aggregation and assisting with membrane insertion [34,36,37].

### 3.2. Fatty Acid α-Oxidation and β-Oxidation

During fatty acid oxidation, peroxisomes only shorten the fatty acid chain (Figure 2). Then the modified fatty acids must be sent to mitochondria for further metabolism because peroxisomes lack Krebs cycle enzymes [14,25,26,29]. VLCFAs are directly oxidized through peroxisomal β-oxidation enzymes. In contrast, fatty acids, which are more hydrophilic than VLCFAs, such as BCFAs, must first undergo peroxisomal α-oxidation before being further metabolized via peroxisomal β-oxidation [11,14,26,29,34,37].

The peroxisomal β-oxidation process consists of four consecutive stages within peroxisomes for 2-carbon chain-shortening and formation of a new acyl-CoA molecule. It is catalyzed by flavin adenine dinucleotide (FAD) containing peroxisomal acyl-coenzyme A oxidase 1 (ACOX1), generating H_2_O_2_ as a byproduct [11,12,14]. Then, hydration by multifunctional protein 1 (MFP1) followed by dehydrogenation by MFP2. Finally, thiolytic cleavage by 3-oxoacyl-CoA thiolase (peroxisomal thiolase) or acetyl-Coenzyme A acyltransferase 1 (ACAA1). Short-chain acetyl-CoA (a molecule that is reduced by two carbon atoms) is further metabolized in the mitochondria after being transported by carnitine. Additionally, this process yields long-chain acyl-CoA as a byproduct, which reenters the β-oxidation metabolic pathway or serves as a substrate for plasmalogen synthesis through peroxisomal lipid biosynthesis [11,14,26].

BCFAs like pristanic and phytanic acids require α-oxidation prior to β-oxidation. For instance, phytanic acid is converted to phytanoyl-CoA and then hydroxylated by PHYH (phytanoyl-CoA hydroxylase) before entering the β-oxidation pathway [11,28,37]. In the metabolic pathway of peroxisomal β-oxidation, pristanoyl-CoA is oxidized by ACOX2 and then undergoes hydration and dehydrogenation by MFP1 and MFP2. Ultimately, the cleavage process is carried out by sterol carrier protein x (SCPx) [12].

As a crucial enzyme in peroxisomal β-oxidation, ACOX1 primarily breaks down long and medium straight-chain fatty acids, both saturated and unsaturated. In contrast, ACOX2 strongly prefers branched-chain fatty acids (BCFA). Meanwhile, ACOX3 has been identified for the degradation of BCFA [29]. In addition, peroxisomal α and β-oxidation are involved in further lipid and non-lipid metabolic pathways, encompassing purine and polyamine metabolism, detoxification of glyoxylate, retinoids, oxysterol derivatives, D-amino acid metabolism, and xenobiotics like ferritin [9,11,12,19,29].

### 3.3. Cholesterol Synthesis

Cholesterol is synthesized in three different organelles. The cholesterol esterification occurs in the ER. The oxidation and conversion of cholesterol to steroids and bile acids occur in the mitochondria and peroxisomes [20]. Lysosomes are known to participate in the transfer of free cholesterol to peroxisomes through synaptotagmin 7 (Syt7), helping to maintain proper cholesterol homeostasis [14]. Although cholesterol synthesis does not primarily occur in peroxisomes, disruptions in peroxisome function hinder cholesterol synthesis [38,39]. It is suggested that peroxisomes have a crucial role in regulating cholesterol balance [20,39].

The process of cholesterol synthesis in peroxisomes consists of two steps catalyzed by the HMG-CoA synthase (HMGCS) and by the HMG-CoA reductase (HMGCR) (Figure 2), then by the enzymes mevalonate kinase (MVK) and phosphomevalonate kinase (PMVK). With the assistance of the farnesyl diphosphate synthase (FDPS) enzyme, isopentenyl pyrophosphate is catalyzed into farnesyl diphosphate. The farnesyl diphosphate is transferred to the ER to proceed to the next stage of cholesterol synthesis. Finally, cholesterol is transported back to the peroxisomes as a precursor for the synthesis of bile acids [20,38,39].

### 3.4. Bile Acid Synthesis

Peroxisomes are essential in synthesizing bile acid precursors like dihydroxycholestanoic acid (DHCA) and trihydroxycholestanoic acid (THCA) (Figure 2), relying on peroxisomal α- and β-oxidation enzymes [14,25,26,29]. A decrease in bile acid intermediates could likely lead to disruptions in peroxisomal α- and β-oxidation function [29,37]. DHCA/THCA-CoA, in its (R) form, requires catalysis by the enzyme 2-methyl-acyl-CoA racemase to convert it into the (S) form. Following this step, the substrate is ready for metabolism through peroxisomal β-oxidation enzymes [11,25,29,37]. This process links peroxisomes to cholesterol clearance, gut–liver axis regulation.

### 3.5. Plasmalogen Synthesis

Ether phospholipids which are crucial for biophysical properties of membranes, act as protectors against oxidative stress and safeguard membranes from the detrimental effects of lipid peroxidation [9,40]. In humans, ether phospholipids comprise about 18% of the total phospholipid mass [12]. They have two typical kinds of bonds, ether bonds and vinyl ether or plasmalogen bonds. Plasmalogens are a special class of glycerophospholipids built upon a glycerol backbone similar to that of triglycerides, but with distinct substitutions at the sn-1 and sn-2 positions. This structural foundation allows diverse fatty acid attachments that define their biological roles. The bonds of plasmalogens have two positions: the first is the sn-1 position comprising palmitic acid (C16:0), stearic acid (C18:0), and oleic acid (C18:1). The second is the sn-2 position, such as PUFA, characterized by head group ethanolamine (PE) found in brain tissue and the head group choline (PC) found in heart muscle. Plasmalogens are also present in moderate amounts in various body tissues and organs, such as the kidneys, skeletal muscles, spleen, and blood cells. The liver contains only a tiny amount of plasmalogens [41,42]. Plasmalogens possess antioxidant activity for trapping ROS [37,41].

The initial stage in plasmalogen biosynthesis in peroxisomes begins with the conjugation of dihydroxyacetone phosphate (DHAP) with a long-chain acyl-CoA ester, transforming it into acyl-DHAP, catalyzed by Glyceronephosphate O-Acyltransferase (GNPAT). The distinctive ether bond at the sn-1 position of ether phospholipids is formed by substituting the sn-1 fatty acid with long-chain fatty alcohol synthesized by fatty acyl-CoA reductase 1/2 (FAR1/FAR2). This reaction is catalyzed by alkylglycerone phosphate synthase (AGPS), producing alkylglycerol-3-phosphate (alkyl-G-3P), which is then transferred to complete the synthesis of plasmalogens in the ER (Figure 2) [14,34,41,42].

Fatty acids in the form of acyl-CoA and fatty alcohols serve as the primary substrate for plasmalogen synthesis in peroxisomes. It is well established that acyl-CoA enters peroxisomes by the peroxisomal ABCD transporter, whereas the mechanism for the entry of fatty alcohol remains less understood [14]. Morita & Imanaka, 2012 [37] reported that dysfunction of ABCD1 reduces plasmalogen levels in the central nervous system.

### 3.6. Polyunsaturated Fatty Acid Metabolism

PUFAs are a subset of LCFA that include arachidonic acid (AA) C20:4, eicosapentaenoic acid (EPA) C20:5, and docosahexaenoic acid (DHA) C22:6 [43]. AA produces prostaglandins, leukotrienes, and thromboxanes, which are pro-inflammatory precursors. AA also generates lipoxins, which possess anti-inflammatory properties. EPA and DHA serve as precursors for resolvin, protectin, and maresin, which are crucial in reducing inflammatory responses, particularly those induced by TNF-α, cyclooxygenase-2 (COX-2), IL-6, and IL-12 [13,43]. In most cases, the metabolism of PUFAs by peroxisomal β-oxidation enzymes is similar to that of VLCFA [9,11,19,44].

Initial PUFA synthesis occurs in the ER, involving both elongation and desaturation. However, very long-chain polyunsaturated fatty acids (VLC-PUFA) precursors cannot be entirely processed by mitochondria and instead rely on peroxisomes via peroxisomal β-oxidation to reduce the 2-carbon chain length. PUFA metabolism also depends on peroxisomal transporters to facilitate entry into peroxisomes [45].

## 4. Peroxisomal Antioxidative Enzymes Are Responsible for Scavenging ROS and RNS

Peroxisomes are both a source and a sink for ROS and RNS, reflecting their dual role in cellular redox regulation. They increased under pathological conditions such as cancer, diabetes, and atherosclerosis. Peroxisomal dysfunction disrupts redox homeostasis, leading to uncontrolled ROS/RNS accumulation and oxidative damage. Under these circumstances, peroxisomal antioxidative enzymes become crucial [11,13,25,26]. ROS and RNS cause cell damage through various mechanisms, including lipid peroxidation, protein oxidation, and DNA damage. These molecules also amplify inflammation by promoting pro-inflammatory cytokine release. ROS species include oxygen radicals like superoxide anion (O_2_^−^), hydroxyl radical (•OH), and non-radical species such as hydrogen peroxide (H_2_O_2_) and peroxynitrite. RNS radicals include nitric oxide (NO•) [37,40,46].

ROS and RNS metabolites are byproducts of oxidation processes within cellular environments. Hydrogen peroxide is generated by peroxisomal enzymes like acyl-CoA oxidases, urate oxidase, xanthine oxidase, D-amino acid oxidase, D-aspartate oxidase, pipecolic acid oxidase, sarosine oxidase, polyamine oxidase, and L-α-hydroxy acid oxidases [37,40,46]. Xanthine oxidase not only generates hydrogen peroxide as a by-product but also produces superoxide anions [40]. Nitric oxide is synthesized by nitric oxide synthase. Superoxide anions are generated exclusively from hypoxanthine and xanthine substrates, while nitric oxide is solely synthesized from L-arginine. The mechanism of ROS-induced modifications in ion transport pathways involves oxidation of sulfhydryl groups located on the ion transport proteins, peroxidation of membrane phospholipids, and inhibition of membrane-bound regulatory enzymes and modification of the oxidative phosphorylation and ATP levels [37,40,46]. Oxidative stress has been demonstrated to cause morphological alterations in peroxisomes, including proliferation and elongation [40].

To counteract oxidative stress, peroxisomes express antioxidative enzymes, including Catalase (CAT), which decomposes H_2_O_2_ into water and oxygen (Figure 2), glutathione peroxidase (GPX), and peroxiredoxin 5 (PRX5), which metabolizes hydrogen peroxide substrates. Other antioxidative enzymes, such as Cu/Zn-superoxide dismutase and Mn-superoxide dismutase, decrease the levels of superoxide anions [16,37,40,46]. Based on Han et al., 2001 [47], nitric oxide can be degraded by enzymes like CAT, horseradish peroxidase, and myeloperoxidase, but not by superoxide dismutase (SOD). Other studies indicate that PRX5 breaks down hydrogen peroxide and also peroxynitrite (ONOO-) [37], which are potent oxidants formed by the reaction between nitric oxide and superoxide anions [40]. These antioxidative mechanisms are essential in macrophages, where tightly regulated ROS/RNS signaling governs inflammation, phagocytosis, and polarization.

## 5. Peroxisome Degradation

Peroxisomes have an approximate lifespan of two days, while dysfunctional organelles degrade more rapidly. Recycling peroxisomes is crucial for maintaining environmental homeostasis, particularly during inflammation and oxidative stress [32,48,49]. There are at least three major pathways by which mammalian peroxisomes are degraded: pexophagy, autolysis (15-Lipoxygenase-Mediated), and Lon protease system [50].

Mammalian peroxisomes primarily utilize three types of pexophagy: macropexophagy, micropexophagy, and chaperone-mediated autophagy (CMA). The CMA specifically targets cytosolic proteins [51], and while selective organellar micropexophagy has not been conclusively proven in mammals [52], macropexophagy is typically considered the primary, and possibly the sole, pathway for organelle degradation in mammalian cells [53].

Autolysis is a process in which 15-lipoxygenase (15-LOX) disrupts the peroxisomal membrane, causing the peroxisomal contents to leak into the cytosol where they are degraded by cytosolic proteases [53,54]. In contrast, the Lon protease pathway, mediated by the peroxisomal Lon protease (LONP2), selectively degrades individual matrix proteins within peroxisomes rather than the entire organelle. This system helps maintain protein quality and can reduce peroxisome size as needed [50,53].

## 6. Peroxisome Proliferator-Activated Receptors (PPARs) in Peroxisomal Gene Expression

PPARs are a group of ligand-activated nuclear hormone receptors that were identified in rodents in 1990 and that serve as transcription factors. PPARs comprise three subtypes: PPARα, PPARβ/δ, and PPARγ. All are involved in a variety of important cellular metabolisms like regulating differentiation, development, apoptosis, and inflammation, moreover they are regulators of lipid, lipoprotein, and carbohydrate metabolism, as well as glucose homeostasis [16,21,22,23,26,55,56,57,58]. Their presence influences peroxisomal abundance and metabolism regulators, such as peroxins, peroxisomal β-oxidation enzymes, and peroxisomal antioxidative proteins genes [9,12]. It has been demonstrated in murine alveolar macrophages that PPARs inhibit the activation of inflammatory response proteins like TNF-α, IL-2, IL-6, IL-8, and metalloproteases by negatively interacting with NF-κB, signal transducer and activator of transcription (STAT), and activator protein 1 (AP-1) signaling pathways. In monocytes and macrophages, PPARs modulate the inflammatory response [56]. Structurally, PPARs resemble steroid or thyroid hormone receptors and are activated by small lipophilic ligands. They share the typical domain structure found in other nuclear receptor family members, featuring an amino-terminal activation function 1 (AF-1) transactivation domain, followed by a DNA-binding domain, and a dimerization and ligand-binding domain that contains a ligand-dependent transactivation function AF-2 at the carboxy-terminal region [22,59].

PPARα influences the balance of ROS and RNS by inducing the expression of peroxisomal antioxidative enzymes like thioredoxin [40], peroxisomal biogenesis proteins [11,26], peroxisomal ABCD transporters, and peroxisomal β-oxidation enzymes [11,16]. Activation of PPARα in murine alveolar macrophages inhibits NF-κB signaling, leading to reduced production of inflammatory cytokines in various cell types. Transcription of genes regulated by PPARα is stimulated during the influx of fatty acids, leading to activation of oxidation systems. This results in increased energy expenditure and reduced fat storage. In contrast, a decrease in PPARα activation leads to reduced oxidation of fatty acids, which ultimately contributes to steatohepatitis and hepatic steatosis during overnight or prolonged fasting [56,57,60].

PPARγ is perhaps the most extensively studied PPAR [56,57,60,61]. Lodhi and Semenkovich, 2014; Aegerter et al., 2022; and Schneider et al., 2014 [26,62,63] revealed that PPARγ plays a crucial role in regulating the expression of genes involved in peroxisomal biogenesis and fatty acid β-oxidation in murine and human alveolar macrophages. Studies in murine and human have shown that PPARγ ligands suppress the transcription of specific pro-inflammatory genes and increase the expression of genes related to anti-inflammatory effects and tissue repair [55,58,62,63,64,65]. PPARγ exists in two isoforms, with PPARγ2 predominantly expressed in adipose tissues, while PPARγ1 has a more widespread expression pattern [22,23].

PPARβ/δ, while not as extensively studied as PPARα and PPARγ [60], shows considerable functional similarities to PPARα but has a broader expression profile [26]. Activation of PPARβ/δ in humans has been shown to counteract metabolic syndrome-related abnormalities without increasing oxidative stress [60,66]. By coordinating peroxisomal gene expression with lipid handling and redox signaling, PPARs serve as central regulators of immunometabolism in macrophages.

## 7. Peroxisomal and PPAR Functions in Organ-Resident Macrophages

**Figure 3:**
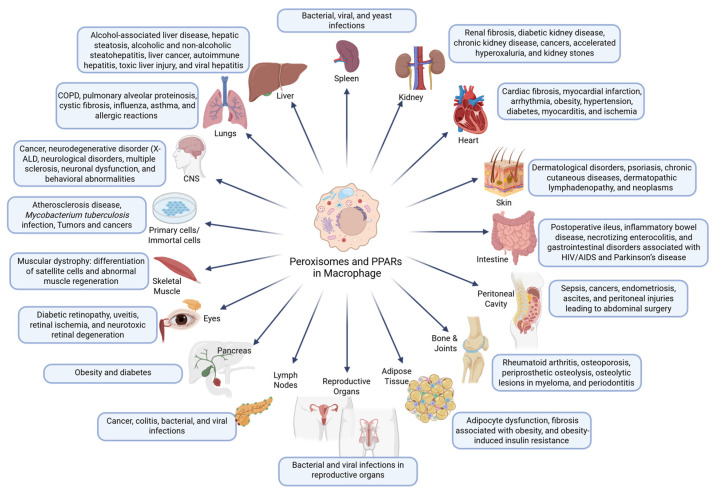
The Role of Peroxisomes and PPARs in Organ-Resident and Cultured Macrophages is Crucial for Disease Pathogenesis. Abbreviations: COPD (chronic obstructive pulmonary disease), X-ALD (X-linked adrenoleukodystrophy), HIV/AIDS (human immunodeficiency virus/Acquired Immune Deficiency Syndrome). Created in BioRender. Wihadmadyatami, H. (2025) https://BioRender.com/25pg3nj (accessed on 10 December 2025).

Tissue-resident macrophages perform unique and context-specific roles depending on their anatomical niche. While all macrophages share core metabolic and immunological machinery, their reliance on peroxisomes and PPAR signaling can differ markedly between tissues. The essential contribution of peroxisomes and PPARs in organ-resident and cultured macrophages, particularly in relation to disease pathogenesis, is depicted in Figure 3. An overview of different organ-resident macrophages and their roles in relation to peroxisomes and PPARs is presented in Table 1.

### 7.1. Central Nervous System (CNS)

Microglia are the typical macrophages that reside permanently in the brain and spinal cord. They play a crucial role in maintaining nervous system homeostasis by supporting brain development, suppressing inflammation, repairing tissue damage, and being involved in the phagocytosis of myelin debris, triggering the recruitment of oligodendrocyte precursor cells, and apoptotic cells. Therefore, microglia are essential in preventing various diseases, such as cancer and neurodegenerative disorders [67,68,118,119]. A deficiency or dysfunction of peroxisomes in microglia can lead to the development of X-ALD [67,68], neurological disorders [120], multiple sclerosis [8], neuronal dysfunction and behavioral abnormalities [69]. The CNS also contains border-associated macrophages (BAMs), such as perivascular, meningeal, and choroid plexus macrophages. Primarily, these macrophages play roles in vascular homeostasis, immune surveillance at the brain border, and maintaining barrier function [121,122].

In people with peroxisomal brain disorders, impaired β-oxidation in microglia results in the inability to break down VLCFA [123]. The absence of *Mfp2* in murine microglia triggers enhanced microgliosis [69,70], resulting in clinical neuropathology, disrupted neuronal function, as well as impairments in behavior, motor skills, and locomotion [69]. Moreover, Beckers et al., 2019 [120] reported that prolonged absence of *Mfp2* in murine microglia results in dysregulation and promotes inflammatory agents.

Raas et al., 2023, Raas et al., 2019 and Tawbeh et al., 2025 [7,71,124] highlight that the deletion of *Abcd1*, *Abcd2*, and *Acox1* in murine microglia cell lines leads to impaired regulation of lipid metabolism, such as accumulation of VLCFA, lipid droplets, lipid inclusions, and modifications of oxysterol levels as well as triggers oxidative stress. In particular, the loss of *Acox1* results in increased expression of IL-1β triggering receptor expressed on myeloid cells 2 (Trem2), which governs microglial polarization, phagocytosis, and the inflammatory response. The disruption in redox balance caused by *Acox1* deletion activates *Pparα*, leading to elevated CAT production, accumulation of neutral lipids (mainly cholesteryl esters), an increased number of peroxisomes and mitochondria, and a reduction in mitochondrial size [67].

This is further supported by the increased expression of *Pparβ/δ* and *Pparγ* [7]. Specifically, the deletion of *Abcd1* and *Abcd2* in microglia leads to elevated levels of AA and DHA, which are known as precursors of signaling lipids that are neuroprotective. Ultrastructural analysis of microglia cell line BV-2 lacking *Abcd1* and *Abcd2* reveals the presence of lipid inclusions similar to those observed in macrophages from X-ALD patients. These lipid inclusions are suspected to result from the accumulation of cholesterol and neutral lipids. Peroxisomal impairment caused by *Abcd1* and *Abcd2* deficiencies has been reported to induce mitochondrial dysfunction and ER stress. Consequently, fatty acid elongation in the ER and mitochondrial fatty acid oxidation may be affected, leading to smaller mitochondria and an increased mitochondrial number [71]. This demonstrates that ABCD1 and ABCD2 play a crucial role in regulating lipid metabolism, thereby ensuring that processes such as FAO and the synthesis of cholesterol, bile acids, plasmalogens, and PUFAs proceed without disruption.

The absence of *Abcd1* in murine microglia cell lines disrupts plasticity, preventing anti-inflammatory responses and ultimately leading to increased myelin damage. The *Abcd1* mutation in murine microglia cell lines results in progressive axonopathy of the spinal cord along with demyelination, referred to as adrenomyeloneuropathy [68]. Gong et al., 2017 [72], revealed that mutations in *Abcd1* with LPS stimulation within spinal cord of humans and mice microglia lead to an increase in pro-inflammatory biomarkers such as IL-1β, TNF-α, NOS2, and COX2. Interestingly, *Abcd1* deletion enhances the phagocytic activity of microglia in mice, increasing the engulfment of phosphatidylserine (PS)-exposing apoptotic neurons and axons, as well as targeting viable neurons and axons. Moreover, it interferes with phospholipid synthesis in microglia, specifically impairing the production of lysophosphatidylcholine (LPC) [72]. *ABCD1*, *ABCD3*, *PEX1*, *PEX5L*, *PEX11β*, *MFP2*, and *CAT* are downregulated in human microglia following TNF-α treatment. However, in the absence of TNF-α treatment, the protein levels of ABCD3 and CAT remain significantly elevated. The reduction in immunolabeling of peroxisomal markers can also be prevented by administering 4-PBA as a peroxisome proliferator [8].

Although tumor-associated macrophages are well known for their roles in cancer progression, their relevance within the CNS and the contributions of CNS-resident macrophages to cancer share similar overarching functions, particularly in supporting glial tumor development and regulating inflammation through mechanisms involving peroxisomes and PPARs [25,125,126]. Peroxisomes and PPARs help microglia cope with lipid-rich, oxidative tumor microenvironments; increased peroxisomal capacity can enable microglial survival and functional reprogramming in situ [126].

Currently, there is no specific information available regarding the roles of peroxisomes and PPARs in BAMs. However, Taketomi & Tsuruta, 2023 [122] recently reviewed that PPAR-γ is one of the key signaling molecules involved in perivascular and border-associated macrophages in both healthy and ischemic brains.

### 7.2. Lungs

Alveolar macrophages, also known as dust cells, are macrophages that reside in the alveoli of the lung. They play a crucial role in innate immunity, maintaining pulmonary organ homeostasis, and act as a key player in balancing defense against pathogens, pollutants, allergens, and toxins, as well as tolerance towards harmless stimuli [2,127,128]. As the primary defense against various pathogens entering the respiratory tract, these cells play a crucial role in eliminating intracellular pathogens by producing ROS, RNS, pro-inflammatory and anti-inflammatory cytokines, and repair mediators, inducing autophagy, and clearing apoptotic cells by phagocytosis (efferocytosis) [2,15,62,73,127,129]. The lungs have two other types of resident macrophages, interstitial macrophages, which are found in the alveolar interstitium, and bronchial interstitial macrophages, which are located within the bronchial interstitium [2,130]. However, alveolar macrophages constitute the majority of lung-resident macrophages, making up around 95% of the total population [130]. Impairment or dysfunction of lung-resident macrophages can lead to various respiratory conditions, including chronic obstructive pulmonary disease (COPD) [2,58], pulmonary alveolar proteinosis, cystic fibrosis, influenza, asthma, and allergic reactions [2].

The importance of lung-resident macrophages in mitigating inflammation cannot be separated from the presence of peroxisomes. Peroxisomes are abundant in alveolar macrophages [9,10]. Karnati & Baumgart-Vogt, 2008 [9] and Wei et al., 2025 [73] reported increased PEX14 expression in human and murine alveolar macrophages. The presence of peroxisomal biogenesis proteins PEX5 and PEX14 has been shown to reduce excessive activation of the inflammasome, prevent the degradation of peroxisomes through pexophagy, alleviate lung inflammation and fibrosis, and prevent lipotoxicity and maintain mitochondrial fitness, thereby supporting the homeostasis, survival, and bioenergetic capacity of alveolar macrophages. This support of peroxisomes is crucial for promoting alveolar regeneration after severe viral injury [73,74]. *Pex5* deletion in mice has been shown to reduce the survival of alveolar macrophages due to increased sensitivity to lipotoxicity [74]. ROS-induced alveolar macrophages release lipid mediators, including leukotrienes and prostaglandins. The upregulated PEX14 can reduce COX-2, a precursor in the production of prostaglandin and pro-inflammatory cytokines such as TNF-α, IL-6, and IL-12, thereby preventing inflammation [10].

Lipid transporter ABCD3 is relatively abundant in alveolar macrophages [9,73,74], likewise ACOX1 [9] and ACAA1 [74]. Alveolar macrophages after viral infection reduced ABCD3 [73]. During viral infection, increased inflammatory activity, mitochondrial damage, and peroxisomal deficiency lead to the accumulation of ROS in alveolar macrophages. During this oxidative stress, the levels of peroxisomal antioxidative enzymes such as Catalase and glutathione peroxidase rise. Suppressing ROS to maintain cellular balance [74,131].

Alveolar and interstitial macrophages are activated and undergo differentiation through the influence of PPARγ [2,63,128,130]. Which is also responsible for regulating the transcriptional program essential for cellular differentiation and function [130]. A decrease in PPARγ levels leads to heightened inflammatory responses, apoptosis, and downregulation of genes involved in lipid peroxidation. However, the induction activity of PPARγ promotes accelerated fibrotic tissue remodeling [9,63], and prevents lung injury [132]. Huang et al., 2019 [133] further reported that influenza A virus infection downregulates *Pparγ* expression in murine macrophages. *Pparγ* has been shown to limit pulmonary inflammation and support host recovery following respiratory viral infection, in part by promoting tissue repair.

### 7.3. Liver

Liver-resident macrophages, commonly known as Kupffer cells, are predominantly located in the liver sinusoids. They play a vital role in maintaining liver homeostasis and contribute to the development of acute and chronic liver injuries [75,134,135]. While some sources reference monocyte-derived liver macrophages found in the portal triad regions of the liver, as well as liver capsular macrophages that emerge during bacterial infections [135,136], Kupffer cells are the most abundant tissue macrophages. They make up 80–90% of the total population of tissue macrophages [135].

Kulle et al., 2022 [2] emphasized that hepatic macrophages play a vital role in monitoring the gut–liver axis for pathogens and toxins. They are responsible for clearing cellular debris and metabolites, aiding liver tissue repair. In addition, they help to maintain iron balance by phagocytosing aged red blood cells and hemoglobin-containing vesicles. Furthermore, these macrophages are involved in bile acid metabolism and the regulation of cholesterol levels by ingesting and transferring low-density lipoprotein-derived cholesterol to hepatocytes [2,76,137]. Several diseases associated with defects in Kupffer cells include alcohol-associated liver disease [75], hepatic steatosis, alcoholic and non-alcoholic steatohepatitis, liver cancer [2,76], cirrhosis [76], autoimmune hepatitis, toxic liver injury, and viral hepatitis [2].

The crucial role of Kupffer cells in mice during inflammation and oxidative stress was reported by Spolarics & Wu, 1997 [77]. Their findings revealed that glutathione reductase and Catalase can detoxify ROS. Even though following LPS or TNF-α induction, the Catalase activity was found to decrease. Kupffer cell damage interferes with regulating inflammation and oxidative stress control [75]. However, activation of PPARα can suppress cytokine accumulation produced by Kupffer cells in mice. This suggests that upregulation of *Pparα* helps protect the liver from oxidative stress and cellular damage via fatty acid oxidation [78,79]. PPARγ has been identified as a major regulator of Kupffer cell polarization. Its interaction with NF-κB plays a role in generating both M1 and M2 phenotypes, as well as in regulating lipid metabolism [76,80,81]. Ni et al., 2022 [81] noted that PPARγ plays a crucial role in resolving inflammation, promoting fibrogenesis, regulating insulin sensitivity, and maintaining glucose homeostasis.

### 7.4. Spleen

Splenic-resident macrophages play a uniquely specialized role among organ-resident macrophages, primarily responsible for iron metabolism and erythrophagocytosis. Impairment of their function may result in anemia and iron accumulation within atherosclerotic plaques [138]. Four distinct types of macrophages have been identified in the spleen, red pulp macrophages, white pulp macrophages (tingible body macrophages), marginal zone macrophages, and marginal metallophilic macrophages.

These diverse splenic macrophages collaborate to address blood-borne infections and initiate effective immune responses. Red pulp macrophages and white pulp macrophages primarily serve as scavengers for senescent erythrocytes, oxidize iron, recognize the capsular polysaccharide glucuronoxylomannan from *Cryptococcus neoformans* and *Streptococcus pneumoniae* under conditions of splenomegaly [139,140,141]. On the other hand, marginal zone and marginal metallophilic macrophages exhibit a robust capacity to internalize pathogens present in the bloodstream, including yeast, bacteria (*Listeria monocytogenes*, *Mycobacterium tuberculosis*, *Escherichia coli*, *Salmonella pneumoniae*, and *Salmonella typhimurium*), and viruses (human immunodeficiency virus, West Nile virus) [139,142,143].

As of now, there is no detailed information available regarding the role of peroxisomes in the spleen. However, it has been reported that PPARγ plays a regulatory role in splenic-resident macrophages in mice. These macrophages are involved in managing inflammation, ROS accumulation, lipid metabolism, iron recycling, and ferroptosis [82].

### 7.5. Kidneys

Renal macrophages play crucial roles in developing inflammation, regulating lipid metabolism, and reducing oxidative stress during acute and chronic kidney injury [144,145]. They monitor and clear urine particles to prevent tubular obstruction [146] and assist in detoxifying uremic toxins [147,148]. The loss or dysfunction of renal macrophages has been associated with various diseases, including renal fibrosis [83,144,145], diabetic kidney disease [149], chronic kidney disease, cancer [83], accelerated hyperoxaluria, and kidney stones [146]. Renal macrophages are classified based on their location within the kidney. These include renal medulla macrophages or juxtatubular macrophages, which are found in the renal medulla [146], glomerular macrophages, which reside in the glomeruli, and interstitial macrophages, which are located in the kidney’s interstitial space [150].

The kidney contains the highest density of peroxisomes. Research by Weng et al., 2014 [151] demonstrated that the deletion of *Pex11*α in proximal tubule cells alters peroxisome abundance and morphology, exacerbates renal lesions and fibrosis, promotes macrophage infiltration, impairs antioxidant capacity, and increases the production of reactive oxygen species. This indicates that the presence of *Pex11*α is crucial for proper kidney function. The role of peroxisomes in kidney-resident macrophages is evident through their involvement in regulating fatty acid oxidation, scavenging ROS, controlling inflammation [83], detoxifying uremic toxins, and preventing the formation of kidney stones [147,148], fostering a pro-angiogenic environment in both healthy and chronically ischemic kidneys [152].

When ureteral obstruction occurs, the kidney experiences inflammation that can ultimately lead to renal fibrosis. This inflammatory process is characterized by an increase in the M1 phenotype associated with elevated levels of pro-inflammatory cytokines. However, the presence of PPARα can decrease the levels of the M1 phenotype and encourage the proliferation of the M2 phenotype in murine models, thus facilitating the resolution of inflammation [84]. Interestingly, inhibiting PPARα and peroxisomal proteins such as superoxide dismutase and Catalase leads to oxidative stress and lipid accumulation [83]. Activating PPARγ effectively prevents the onset of chronic dysfunction in the kidneys [85].

Excessive ROS generated by hyperglycemia in diabetes mellitus stimulates macrophage polarization toward the M1 phenotype, triggering the release of inflammatory factors. The process further escalates PRDX2 production and secretion in kidney cells [149]. Similarly, the accumulation of uremic toxins in patients with chronic kidney disease or atherosclerosis leads to macrophage activation, resulting in increased production of pro-inflammatory cytokines and the induction of oxidative stress [147,148]. The presence of peroxisomes offers a solution by helping to reduce inflammation and regulate oxidative stress through activating PPARs [83,84,85] and peroxisomal antioxidative enzymes [149,151].

### 7.6. Heart

Cardiac-resident macrophages make up about 6% to 10% of heart tissue [153,154]. They play a crucial role in the development and progression of cardiovascular diseases such as cardiac fibrosis, myocardial infarction [155,156], arrhythmia, obesity, hypertension, diabetes, myocarditis, and ischemia [155,157]. They perform various functions, including regulating inflammation, facilitating cardiac remodeling, and clearing cellular debris [155,156,158], modulating arterial tone, patrolling blood vessels, remodeling heart valves, supporting osmoregulation, and contributing to electrical conduction [155]. Qin et al., 2024 [154] reported that macrophages are more abundant in the ventricles compared to the atria. This difference is likely due to the macrophages’ role in clearing cellular debris and repairing damaged tissue, ensuring that the blood pumped throughout the body remains clean and safe. So far, there is no direct evidence confirming the role of peroxisomes in cardiac-resident macrophages. However, given their established functions in regulating inflammation, maintaining redox balance, and managing lipid metabolism, it is plausible that peroxisomes also contribute to the activity of these cells [153]. Colasante et al., 2015 [35] reported that peroxisomes are abundant in cardiac tissue. However, their findings did not specifically address peroxisomes in cardiac-resident macrophages. Nonetheless, peroxisomal dysfunction in the heart significantly impacts metabolic remodeling and can lead to heart failure.

Although there is no specific information yet regarding the role of peroxisomes in the heart macrophages, several studies have revealed that peroxisomes contribute to cardiac function, for instance, through the synthesis of plasmalogen, which interacts with cardiolipin [159,160]. Disruptions in cardiolipin synthesis have been linked to the development of Barth syndrome [159]. PPARs have been reported to be present in cardiac-resident macrophages, where they play key roles in glucose and lipid metabolism [86]. They also help regulate redox balance by enhancing the transcription of antioxidant-related genes and suppressing the production of ROS. Activation of PPARα reduced the production of ROS and cellular damage following ischemic injury by enhancing the production and activity of peroxisomal antioxidant enzymes. Other research has indicated that activating PPARγ boosts the transcription of antioxidant-related genes, while PPARβ/δ activation suppresses the production and signaling of nitric oxide. Additionally, the deletion of *Pparβ/δ* leads to a downregulation of antioxidant-related genes [87].

### 7.7. Intestine

Intestinal macrophages are classified into several types based on their location in the intestine, including mucosal macrophages [88], lamina propria macrophages, muscularis macrophages [89], perivascular macrophages [161], ileal macrophages, and colonic macrophages [162,163]. Overall, intestine-resident macrophages play crucial roles in preventing infections from pathogens entering the gastrointestinal tract, maintaining immune homeostasis, regulating inflammatory responses, repairing damaged tissues, clearing dead cells and foreign debris, and controlling gut motility and secretion [88,89]. Disruptions in macrophages in the intestine can exacerbate gastrointestinal diseases, including postoperative ileus, inflammatory bowel disease, necrotizing enterocolitis, and gastrointestinal disorders associated with Acquired immunodeficiency syndrome (AIDS) and Parkinson’s disease [89].

Some studies suggest that peroxisomes play a crucial role in intestinal development and repair, particularly in lipid metabolism [164,165]. Peroxisomes play a crucial role in maintaining the integrity of intestinal epithelial junctions. When peroxisomes are dysfunctional, it results in reduced villus size, cell growth defects, and a decreased number of stem cells [166]. Disruption of peroxisomal function also results in elevated cell death and epithelial instability, which in turn alter the intestinal microbiota composition, weaken immune responses in the gut during infections, and negatively affect the survival of the organism [167].

### 7.8. Peritoneal Cavity

Peritoneal macrophages are resident in the peritoneal cavity, which is covered by the peritoneum, and participate in various aspects of innate and acquired immunity. Numerous studies suggest that the immunological and inflammatory responses of peritoneal macrophages are closely associated with pathogenic processes such as sepsis, cancer, endometriosis, ascites, and peritoneal injuries [168,169,170,171]. Resident peritoneal macrophages are able to regulate peritoneal B1 cells and migrate via nonvascular pathways to the liver, ovaries, spleen, pancreas, and intestine [169,171]. Peritoneal macrophages can inhibit T cell proliferation and mitigate inflammation. Consequently, the regulation of peritoneal macrophage functions within the peritoneal cavity emerges as a crucial aspect of inflammatory diseases [170]. There are two types of resident macrophages present in the peritoneal cavity: large peritoneal macrophages (an abundant and long-lived population) and small peritoneal macrophages (a rare and short-lived population) [171,172].

PEX14 is abundantly expressed in murine peritoneal macrophages [10,74], and its presence has been shown to reduce COX-2 significantly [10]. Its presence is believed to be closely related to peroxisomal biogenesis [25]. Peroxisomal ABCD transporters are upregulated expressed in murine peritoneal macrophages. *Abcd1* deficiency causes impaired peroxisomal β-oxidation and leads to the accumulation of VLFCA. Interestingly, peritoneal macrophages which have only half the amount of *Abcd2* compared to *Abcd1* after mutation, do not impair the performance of peroxisomal β-oxidation, nor does it lead to the accumulation of VLCFA during its reduction. However, the loss of both *Abcd1* and *Abcd2* results in a significant increase in VLCFA levels and impaired peroxisomal β-oxidation [90].

MFP2 and ACAA1 have been detected in murine peritoneal resident macrophages [18,74]. These enzymes are key components of peroxisomal β-oxidation playing an active role in fatty acid oxidation. Interestingly, deleting *Mfp2* leads to alterations in VLCFA distribution and reduces pro-inflammatory responses in peritoneal M1 phenotype but not in peritoneal M2 phenotype. *Mfp2* deficiency in the M2 phenotype enhances the expression of the anti-inflammatory gene Retnla. Overall, these findings suggest that although the production of inflammatory chemokines is reduced, the deletion of *Mfp2* in murine macrophages still allows for a normal immune response following acute inflammatory stimulation. The disruption of the inflammatory response caused by *Mfp2* deletion does not impact immune cell infiltration, macrophages continue to activate monocytes and neutrophils. This indicates that MFP2 plays a role in refining macrophage phenotype by influencing lipid profiles during macrophage polarization, even though there is a decrease in lipid quantity and an increase in their size [18].

Activated murine peritoneal macrophages show an increase in *Pparα* and *Pparγ* expression [91,92,93]. This inhibits inducible nitric oxide synthase expression and production of inflammatory cytokines, such as TNF-α, IL-1β, and IL-6 [58,173]. Von Knethen et al., 2011 [174] stated that PPARγ activation is required to induce heme oxygenase-1 (HO-1) and Interferon-β (IFN-β) expression in murine peritoneal macrophages. HO-1 protects cells from oxidant-induced damage during inflammation by breaking down heme into carbon monoxide (CO), biliverdin, and ferrous iron. At the same time, IFN-β facilitates the reprogramming of macrophages during the resolution phase. Furthermore, PPARβ/δ can strongly inhibit lipopolysaccharide (LPS)-induced COX-2 and iNOS transcription in murine peritoneal macrophages [173]. He et al., 2014 [175] reported that LPS-induced murine peritoneal macrophages exhibit increased expression of *Ppar-γ*.

### 7.9. Bone and Joints

Osteoclasts are defined as bone-resident macrophages responsible for bone resorption, bone remodeling [176], and contributing to inflammatory processes [177]. The multinucleated cells release acids and proteolytic enzymes that facilitate the dissolution of minerals and the degradation of bone matrix. Thus, they play a crucial role in diseases such as rheumatoid arthritis, osteoporosis, periprosthetic osteolysis, osteolytic lesions in myeloma, and periodontitis [176,177,178]. Extensive information is available on the role of peroxisomes in bone. Nevertheless, PEX14 and Catalase are the only components that have been specifically identified in osteoclasts [94]. Panagopoulos et al., 2017 [95] reported that peroxisomal antioxidative enzymes, such as peroxidases, suppress the increase in hydrogen peroxide levels during the pro-inflammatory phase. PPARγ has been detected in bone tissue. Its accumulation suppresses osteoblastogenesis while promoting osteoclastogenesis, thereby playing a significant role in inhibiting osteoporosis. Additionally, PPARγ regulates lipid metabolism and modulates inflammation within bone tissue [96].

Synovial macrophages are resident immune cells found on the surface of the synovial membrane in joints, specifically distributed across both the lining and sub-lining layers at the cartilage–pannus junction [179,180]. They secrete regulatory factors contributing to cartilage and bone turnover while clearing cellular debris and pathogens to prevent sterile and septic inflammation. Synovial macrophages play a crucial role in osteoarthritis and synovitis by releasing both pro-inflammatory and anti-inflammatory cytokines [180]. Currently, there is no specific information about the role of peroxisomes in synovial macrophages. However, because peroxisomes are known to be involved in regulating inflammation, it is believed that they may also have a role in this context. Further in-depth research is necessary to confirm this hypothesis.

### 7.10. Adipose Tissue

Adipose tissue macrophages are the most prevalent immune cells in adipose tissue, comprising approximately 10–15% of the total cell population in healthy adipose tissue [181]. They play a central role in adipocyte dysfunction and fibrosis associated with obesity [182,183], and are key contributors to obesity-induced insulin resistance [97,184].

Although expressed at low levels, Matsushita et al., 2025 [74] explained that PEX14 and ACAA1 are present in adipose-resident murine macrophages. They are believed to play a crucial role in regulating lipid metabolism and inflammation. Catalase has been identified in adipose-resident murine macrophages, which play a crucial role in reducing oxidative stress during inflammation. Catalase is also involved in regulating macrophage polarization and enhancing insulin sensitivity. Impaired mitochondrial respiratory capacity has been shown to elevate Catalase activity. Interestingly, Catalase deficiency leads to oxidant-induced tissue damage, increases mitochondrial ROS levels, accelerates macrophage infiltration, and promotes the formation of crown-like structures in adipose tissue-features typically associated with the M1 phenotype [97]. Li et al., 2020 [98] further reported that adipose-resident macrophages produce peroxisomal antioxidative glutathione, which plays a key role in suppressing ROS during inflammation. *Pparγ* has been reported to promote the M2 phenotype in mice. During diet-induced obesity, adipose-resident macrophages shift to a more pro-inflammatory M1 phenotype in mice, characterized by increased inflammatory cytokine production [99].

### 7.11. Reproductive Organs

Reproductive organ-resident macrophages are classified based on sex. In males, testicular macrophages include two main subtypes: peritubular macrophages, which are located near the surface of the seminiferous tubules, and interstitial testicular macrophages, which reside within the interstitial spaces of the testis [100,101]. In females, resident macrophages are classified into ovarian macrophages and uterine macrophages [185].

Testicular macrophages play a crucial role in maintaining testicular homeostasis. They serve as a barrier against bacterial infections such as *Escherichia coli*, *Klebsiella* sp., and viral infections, including HIV-1 and Zika virus [100,101]. Ovarian macrophages play a crucial role in regulating essential processes such as follicular development, ovulation, and luteinization. While uterine macrophages are responsible for endometrial remodeling, promoting immune tolerance, and supporting placentation within the uterus [185].

Peroxisomes have been identified in testis, but they are absent in mature spermatozoa. In the testis, they are known to play a vital role in spermiogenesis and lipid metabolism [186]. Wang et al., 2022 [187] reported that peroxisomes are involved in steroid biosynthesis, follicular growth, oocyte maturation, and ovulation within the female reproductive organs. PPARs have been shown to play a crucial role in female reproduction, influencing ovarian function, pregnancy, and the communication between mother and fetus [188]. PPARα and PPARγ have been identified in murine ovarian macrophages, where they regulate metabolic processes, support ovulation, and function as anti-inflammatory mediators [102].

### 7.12. Lymph Nodes

Lymph node-resident macrophages are classified into several types based on their specific locations within the lymph node tissue, including subcapsular sinus macrophages, medullary sinus macrophages, medullary cord macrophages, and interfollicular macrophages [103]. Subcapsular sinus macrophages are a rich source of resident macrophages found within the lymph nodes. Lymphoid tissue is primarily responsible for trapping and presenting antigens to B cells. In this context, subcapsular macrophages play a vital role in preventing the systemic dissemination of pathogens carried through the lymph. Additionally, they help initiate immune responses by activating various innate effector and adaptive memory cells, including follicular memory T cells and memory B cells, that are either pre-positioned or rapidly recruited to the subcapsular region during infection or inflammation [104,105]. The role of subcapsular macrophages is closely associated with their function as the frontline of lymphatic immune defense against a range of diseases, including those caused by viruses (influenza virus, adenovirus), bacteria (*Staphylococcus aureus*, *Pseudomonas aeruginosa*), parasites (*Toxoplasma gondii*, Malaria), as well as conditions like cancer and colitis [106].

Currently, there is no specific information available regarding the roles of peroxisomes and PPARs in murine macrophage lymph nodes. However, the activities of lymph node–resident macrophages, such as regulating ROS balance, lipid metabolism, and inflammation [103,104,105,106], are closely associated with the roles of peroxisomes and PPARs.

### 7.13. Pancreas

Islet macrophages are responsible for regulating pancreatic vascular remodeling, islet structure remodeling, insulin secretion capacity, glucose homeostasis, lipid metabolism, inflammation control, and the production of connective tissue growth factors [107,189]. Islet macrophages are known to influence the maintenance of pancreatic beta cells. Depletion of islet macrophages impairs islet remodeling, disrupts insulin secretion, delays islet revascularization, and reduces vascular density [107]. In cases of obesity and diabetes, islet macrophages have been consistently reported to increase in number and adopt a more pro-inflammatory phenotype [107,189,190]. Disruption in islet macrophage polarization affects beta cell identity [107]. A study by Chan et al., 2019 [191] reported that the accumulation of the M2 phenotype contributes to beta cell stress and the loss of beta cell identity.

Currently, there is no direct evidence confirming the presence of peroxisomes or PPARs in pancreatic-resident macrophages. Nevertheless, studies have shown that lipid metabolism, inflammation regulation, and oxidative stress take place in islet macrophages [106,107]. Importantly, Bihan et al., 2005 [108] demonstrated that PPARα plays a significant role in regulating insulin secretion and glucose homeostasis in mice. Impairment of PPARα function can disrupt glucose metabolism, potentially leading to obesity, diabetes, and kidney failure [107,189,190]. PPARγ has also been identified in pancreatic stellate cells, which are recognized as major cytokine producers in the pancreas. These cells have been shown to regulate inflammation and phagocytosis within pancreatic tissue [109].

### 7.14. Skin

Skin-resident macrophages consist of two primary types: Langerhans cells, located in the epidermis, and dermal macrophages, which reside in the dermis. These immune cells act as the first line of defense against invading pathogens and are crucial for controlling inflammation, promoting wound healing, regulating lipid metabolism, and maintaining ROS balance in the skin [110,111]. Their roles have been associated with various dermatological disorders, like psoriasis, chronic cutaneous diseases, dermatopathic lymphadenopathy, and neoplasms [111]. A deficiency of Langerhans cells leads to excessive lipid oxidation, which triggers ferroptosis. Mitochondrial dysfunction causes metabolic stress and accelerates the polarization of Langerhans cells toward the M1 phenotype. Langerhans cells lacking autophagy undergo ferroptosis [110].

PPARs are expressed in the skin tissue, particularly in the epidermis, which is an active site for lipid metabolism. Activation of PPARs promotes keratinocyte differentiation and triggers anti-inflammatory responses, thereby helping to reduce inflammation [192]. Specifically, PPARγ plays a key role in regulating various macrophage functions. In the skin, it is essential for guiding wound macrophages to remove apoptotic cells, a process critical for efficient wound healing. This underscores its potential as a therapeutic target for improving skin repair. A lack of *Pparγ* in murine macrophage disrupts the healing process, resulting in decreased collagen deposition, impaired angiogenesis and granulation tissue formation, and elevated levels of pro-inflammatory cytokines [112].

### 7.15. Eyes

Various types of macrophages reside in the eyes, including vitreal macrophages or hyalocytes (the most abundant eye-resident macrophages), microglia located in the ocular nervous system [113], perivascular macrophages resided on postcapillary venules [193,194], and monocyte-derived macrophages situated at the vitreoretinal interface [114]. They play a crucial role in maintaining the balance of inflammation, immune cell migration, and performing erythrophagocytosis in diabetic retinopathy and uveitis [193,195]. Additionally, they are essential for ocular neovascularization, vascular development, and are also involved in the resolution of eye diseases such as retinal ischemia and neurotoxic retinal conditions [113,196,197]. During retinal pathology, there is a reduction in the number of vitreal macrophages [113].

Although specific information about the role of peroxisomes in eye-resident macrophages is still lacking, numerous studies have emphasized the critical functions of these macrophages in lipid metabolism, regulation of inflammation, and ROS scavenging—all of which are processes typically associated with peroxisomal activity. Several peroxisomal antioxidative enzymes, including Catalase, superoxide dismutase, glutathione, and glutathione peroxidase, contribute to ROS detoxification in the eye [198,199]. The accumulation of ROS in ocular tissues is linked to eye diseases such as diabetic retinopathy, age-related macular degeneration, nuclear cataracts, and corneal endothelial dystrophy [199].

Das et al., 2019 [198] reported the presence of peroxisomal biogenesis proteins PEX5 and PEX14, peroxisomal ABCD transporters, peroxisomal α- and β-oxidation enzymes (PHYH, ACOX1, MFP2, SCPx, and ACAA1), and the peroxisomal plasmalogen synthesis enzyme GNPAT in the murine retinal tissue. Furthermore, a recent study, Das et al., 2021 [200] revealed that deleting *Mfp2* disrupts lipid homeostasis in the murine retina, leading to visual dysfunction. Meanwhile, PPARα is an essential transcription factor that regulates lipid metabolism, detected in eye-resident macrophages. Its deletion leads to increased retinal pericyte loss and impaired neuronal function [114].

### 7.16. Skeletal Muscle

Muscle macrophages play a crucial role in maintaining muscle homeostasis, facilitating repair, and promoting regeneration. After injury, they switch from pro-inflammatory to repair phenotypes. These macrophages interact with muscle fibers, satellite cells, and the extracellular environment to regulate inflammation, clear debris, and facilitate muscle regeneration. Disrupted satellite cell differentiation and impaired muscle regeneration contribute to chronic muscle disorders, such as muscular dystrophy, leading to irregular or uncoordinated activation [115].

*Pex5* deficiency in mice impairs mitochondrial function, decreases exercise capacity in skeletal muscle, and accelerates age-related muscle degeneration. Macrophages in muscle depend on peroxisomes to clear toxic lipids or ROS in the muscle environment, especially after injury, when fatty acids and oxidative stress increase. Since macrophages influence mitochondrial behavior in muscle cells, peroxisomes in macrophages might help maintain redox balance and protect both macrophages and surrounding muscle cells from oxidative damage [201].

A key study demonstrated that PPAR-γ in muscle macrophages of mice is essential for proper skeletal muscle regeneration. Specifically, macrophage PPAR-γ regulates the expression of Growth/Differentiation Factor 3 (GDF3), a growth factor that promotes the fusion of muscle progenitor (satellite) cells [116]. PPARs likely help to modulate the inflammatory response during injury or metabolic stress, favoring a pro-repair phenotype, as observed in a murine skeletal muscle cell line [117].

## 8. Peroxisomal and PPARs Functions in Cultivated Macrophages

Macrophages obtained from various primary and immortalized sources display distinct metabolic functions influenced by peroxisomes and PPARs, as summarized in Table 2.

### 8.1. Bone Marrow-Derived Macrophages (BMDMs)

Bone marrow-derived macrophages can be isolated from bone marrow and cultivated in vitro with growth factors. Macrophage colony-stimulating factor (M-CSF) is a lineage-specific growth factor for fostering the proliferation and differentiation of committed myeloid progenitors into cells within the macrophage or monocyte lineage. Mice lacking functional M-CSF exhibit deficiencies in both macrophages and osteoclasts, leading to the development of osteopetrosis [213,214]. Cultivated BMDMs are commonly utilized as a macrophage model in the majority of immunological studies [215]. Several sources report that in vivo BMDMs play an essential role in the formation of tissue-resident macrophages in skin, intestine, lymph, and heart tissue [2,202]. BMDMs are endowed with many peroxisomes.

Our group reported an upregulation of peroxisomal biogenesis proteins (PEX5, PEX11β, PEX13, PEX14, PEX19), peroxisomal membrane transporter ABCD3, peroxisomal β-Oxidation enzymes (ACOX1, MFP2, ACAA1), and peroxisomal antioxidative (Catalase) enzymes during *Mycobacterium tuberculosis* infection in murine BMDMs [15]. During infection, the bacteria interact with the macrophage mannose receptor in BMDMs, modulating peroxisomes to support their survival. At the early stages of bacterial infection, the accumulation of ROS will initiate an increase in peroxin and ABCD3 levels. During this period, peroxisomal β-oxidation leads to further ROS buildup. Concurrently, Catalase and peroxiredoxin transcription are upregulated to eliminate excess ROS, as well as peroxynitrite, organic peroxides [203], cyclooxygenases and prostaglandin [216] after being stimulation with NO, LPS, and IFN-γ [203,216]. The deletion of *Acox1* and *Mfp2* can have severe consequences, including disruptions in lipid metabolism [18]. PPARγ is detected in murine BMDMs [132,204,205,206]. It plays a crucial role as an anti-inflammatory regulator by suppressing NFκB and regulating oxidative metabolism [132,206]. Babaev et al., 2007 [207] found that PPARα exhibits anti-atherogenic effects in murine BMDMs, suggesting its potential as an agent against atherosclerosis disease.

### 8.2. RAW264.7 Cell Line

The RAW264.7 cell line, supplied by the American Type Culture Collection (ATCC), is the most frequently used in vitro cell model for macrophage research over the past 40 years. Originally derived from an Abelson leukemia virus-transformed cell from BALB/c mice, these macrophages are most responsive to LPS induction, producing nitric oxide and enhancing phagocytosis [217].

Peroxisome biogenesis protein PEX14 is abundant in RAW264.7 cells. In vivo, its abundance can decrease pro-inflammatory cytokines like TNF-α, IL-6, IL-12, and COX-2, but promote an increase in the anti-inflammatory cytokine IL-10. Deletion of *Pex14* is able to reverse the top levels of COX-2 and TNF-α proteins [10]. PEX5 and ABCD3 were also detected in RAW264.7 cells. Their levels increase further after infection of the cell line with *Mycobacterium tuberculosis*. This increase in peroxisomes is associated with the interaction between the bacteria and the macrophage mannose receptor, facilitating bacteria’s survival in macrophages [15]. *Mfp2*, which plays a crucial role in the peroxisomal β-oxidation metabolic process, has also been detected in RAW264.7 cells. Its deletion has been shown to elevate pro-inflammatory cytokine levels while reducing anti-inflammatory cytokine expression [10].

Peroxiredoxin, a peroxisomal antioxidative enzyme, was found to be increased in RAW264.7 cells after stimulation with IFN-γ, nitric oxide synthase-2 (NOS2), and LPS. An increase in anti-inflammatory cytokine activity after stimulating peroxiredoxin has been proven to reduce the levels of pro-inflammatory cytokines IL-1β and TNF-α [203,208]. PPARγ has also been reported to be expressed in RAW264.7 cell lines, playing a crucial role in reducing ROS levels [174,209]. Luo et al., 2017 [80] added that PPARγ is actively involved in RAW264.7 cell line polarization and is influenced by lipid levels.

### 8.3. Other Cultivated Macrophage Models

Recently, induced pluripotent stem cell (iPSC)-derived human macrophages (iPSDMs) have emerged as a valuable model for studying peroxisomes. In iPSDMs, *Pex3* deficiency combined with bacterial infection leads to increased oxidative stress. Notably, the levels of peroxisomal proteins, such as PEX14, ABCD3, ACOX1, HSD, and Catalase, were found to be significantly elevated. Furthermore, the expression of over 30 genes encoding peroxisomal proteins and enzymes, as well as *Ppars*, was also observed [210].

PPARs have been identified in macrophages derived from human peripheral blood mononuclear-derived macrophages [66,211] and embryonic stem cells [212]. Mainly, PPARγ facilitates the differentiation of monocytes into the M2 phenotype. In contrast, the role of PPARβ/δ in this differentiation process has only been documented in mice [66]. Although Souissi et al., 2008 [92] previously reported that PPARα has been identified in macrophages derived from human mononuclear cells. Activated PPARγ is capable of enhancing phagocytic activity in the M2 phenotype [56], involved in the development of atherosclerosis [92], and capable of reducing the prostaglandin precursor COX-2 [10]. PPARγ has also been found to have further anti-inflammatory effects, shape macrophage functions, and regulate migration [218].

In Tumor-associated macrophages (TAMs), the PPARs are closely involved in regulating their phenotype, inhibiting the angiogenesis of the tumor, and promoting immunostimulatory activities [61,93]. The presence of PPARα and PPARγ induces differentiation to the M2 phenotype, suppressing T-cell lymphoma. PPARβ/δ in conjunction with PPARγ has also been reported to inhibit NF-κB signaling and STAT1, including CXC chemokine ligand 8 (CXCL8) and CXCL1, while promoting M2 phenotype through the regulation of fatty acid metabolism. Elevated levels of arachidonic acid, linoleic acid, and lipid droplets can activate PPARβ/δ and polarize TAM in breast and ovarian cancers [61,93,219,220].

## 9. Peroxisomes and PPARs Play a Pivotal Role in Macrophage Metabolism

**Figure 4:**
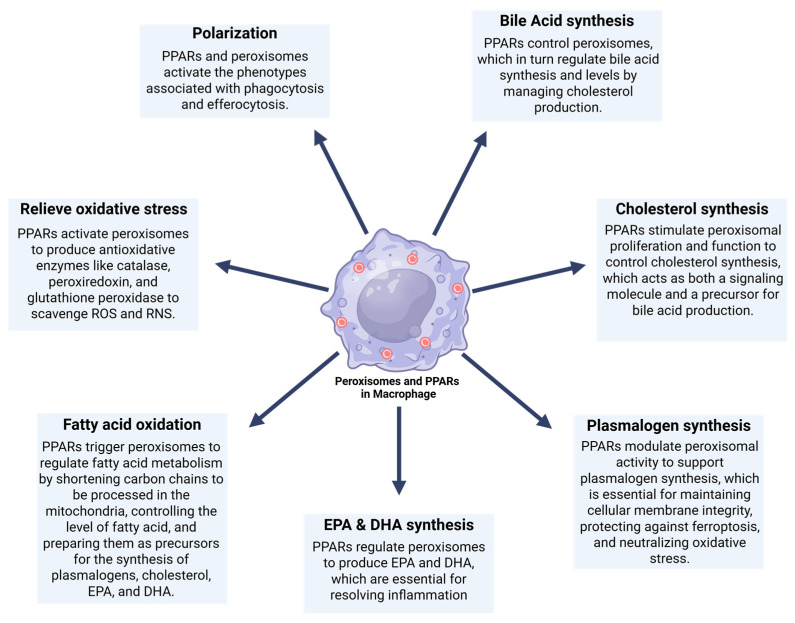
The Role of Peroxisomes and PPARs in Macrophages. Abbreviations: PPARs (Peroxisome Proliferator-Activated Receptors), EPA (Eicosapentaenoic acid), DHA (Docosahexaenoic acid), ROS (Reactive Oxygen Species), RNS (Reactive Nitrogen Species). Created in BioRender. Wihadmadyatami, H. (2025) https://BioRender.com/5mrll4z (accessed on 10 December 2025).

Within macrophage metabolic pathways, peroxisomes and PPARs are essential for managing inflammation, oxidative balance (ROS and RNS), ferroptosis, angiogenesis, and overall cellular homeostasis. Their summarized functions are provided in Table 3 and Figure 4.

### 9.1. Peroxisomes and PPARs Suppress Inflammation and Oxidative Stress and Promote the Resolution of Inflammation

The expression of peroxins such as PEX5, PEX11β, PEX13, PEX14, and PEX19 increases significantly during inflammatory conditions. This increase significantly reduces COX-2 activity and effectively lowers the levels of pro-inflammatory cytokines [15]. The role of peroxins is crucial in peroxisome biogenesis. Disturbances in peroxin expression will impair peroxisome metabolism, subsequently affecting cellular metabolic functions. For example, PEX5 and PEX7 are responsible for trafficking peroxisomal matrix proteins to the organelle, while PEX5 levels influence the activity of peroxisomal antioxidative enzymes, and PEX7 affects the activity of AGPS [17].

In macrophages, PEX5 and PEX7 play a role in phagocytosis, with one of the effects being caused by the absence of functional peroxisomes [225]. PEX13 and PEX14 are entirely hydrophobic and create stable rod-like structures that extend into the cytosol. It possesses only one transmembrane segment [30,31]. These proteins have a multi-tasking role, not only facilitating peroxisomal protein import but also playing a crucial role in peroxisome motility by serving as a membrane anchor for microtubules [226]. In a recent study, it is stated that a decrease in peroxisomes follows the loss of PEX14 [227]. Damage to the PEX14 genes can cause peroxisome biogenesis disorders, including Zellweger syndrome [17]. Moreover, an increase in PEX11β level indicates significantly more peroxisome proliferation, possibly as an adaptive response to inflammatory homeostasis [17].

**Figure 5:**
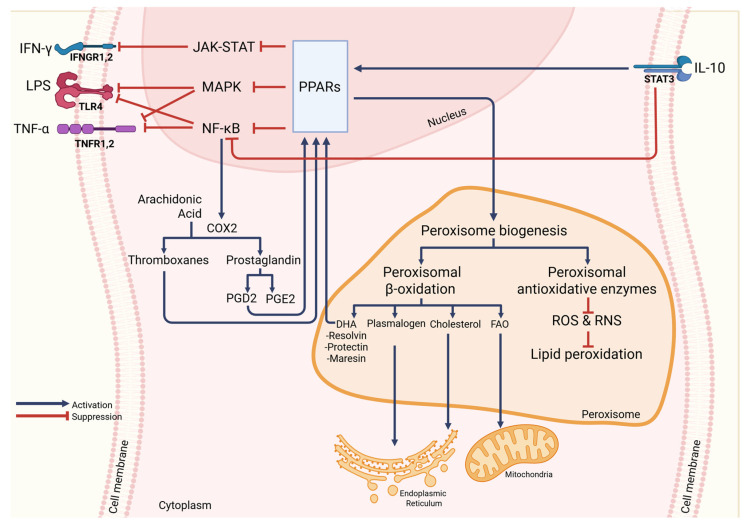
Role of Peroxisomes and PPARs in Anti-Inflammatory Macrophage. The activation of anti-inflammatory cytokines such as IL-10 can stimulate peroxisomes and PPARs, contributing to the resolution of inflammation in macrophages. IL-10 signaling in macrophages operates through the STAT3 transducer to activate PPARs, which in turn suppress NF-κB activity. Additionally, IL-10 signaling via STAT3 transducer is capable of directly inhibiting NF-κB activation. The suppression of NF-κB disrupts downstream signaling triggered by inflammatory stimuli such as LPS, IFN-γ, TNF-α, and PGE2, ultimately leading to reduced inflammatory responses. Furthermore, arachidonic acid is driven to produce increased levels of thromboxanes and PGD2, which serve as natural ligands for PPARs. The enhanced activation of PPARs ultimately promotes the transcription of genes involved in peroxisomal biogenesis, peroxisomal β-oxidation, and the production of peroxisomal antioxidative enzymes. This upregulation contributes to increased synthesis of DHA, which plays a role in supporting the resolution of inflammation. The elevated production of plasmalogens and cholesterol further strengthens cell membrane integrity, signaling, and antioxidant defenses. Efficient regulation of lipid metabolism by peroxisomes also helps prevent lipotoxicity. Moreover, the accumulation of antioxidative enzymes such as Catalase helps restore redox balance by reducing oxidative stress, thereby limiting lipid peroxidation. Abbreviations: IFN-γ (Interferon-γ), IFNGR (Interferon gamma Receptor), JAK-STAT (Janus Kinase-Signal Transducer and Activator of Transcription), LPS (Lipopolysaccharide), TLR4 (Toll-like receptor 4), MAPK (Mitogen-activated protein kinase), TNF-α (Tumor Necrosis Factor-α), TNFR1 (Tumor Necrosis Factor Receptor 1), NF-κB (Nuclear Factor kappa-light-chain-enhancer of activated B cells), PPARs (Peroxisome Proliferator-Activated Receptors), IL-10 (Interleukin-10), STAT3 (Signal Transducer and Activator of Transcription 3), COX2 (Cyclooxygenase-2), PGD2 (Prostaglandin D2), PGE2 (Prostaglandin E2), DHA (Docosahexaenoic acid), FAO (Fatty Acid Oxidation), ROS (Reactive Oxygen Species), RNS (Reactive Nitrogen Species). Created in BioRender. Wihadmadyatami, H. (2025) https://BioRender.com/n4kgirv (accessed on 10 December 2025).

The role of peroxisomes in reducing inflammation is associated with their synthesis of DHA-derived compounds like protectins, resolvins, and maresins (Figure 5). DHA is an anti-inflammatory compound generated by peroxisomal β-oxidation. Even though they also produce inflammation precursors, such as prostaglandins, leukotrienes, and thromboxanes [13]. The production of inflammation-resolving agents is much more significant and plays a crucial role during recovery. Several studies have shown that an abundance of DHA can cut down the accumulation of COX-2 and pro-inflammatory cytokines in macrophages. The presence of Catalase and peroxiredoxin can reduce the accumulation of ROS and RNS during inflammation [13,208,228]. Moreover, they suppress COX-2 and pro-inflammatory cytokine levels. Catalase and peroxiredoxin play a crucial role in regulating the balance of ROS and RNS generated by β-oxidation metabolism in both peroxisomes and mitochondria, as well as the oxidative byproducts of nitric oxide synthase. The production of ROS and RNS can rapidly increase during inflammation, aging, and peroxisomal biogenesis [13,228]. The increased oxidative stress can stimulate PPARγ to signal peroxisomes to produce more antioxidative enzymes to counteract the buildup of ROS and RNS [228].

The presence of PPARγ has been shown to enhance anti-inflammatory cytokines like IL-10 in macrophages induced by LPS/IFN-γ [10,208]. Under anti-inflammatory conditions, the role of peroxisomes becomes even more essential as macrophage metabolism increasingly depends on fatty acid oxidation. This intensifies the significance of peroxisomal enzymes in lipid metabolism. Anti-inflammatory agents such as STAT3 [208], STAT6 [229], and Ym1 [18] are also increased during LPS/IFN-γ stimulation.

Plasmalogen serves as a key component in the formation of biological membranes [230,231,232,233]. An actual example in neurons is its capacity to prevent neuroinflammation, to enhance cognitive function, and to protect against neuronal cell death. The composition of plasmalogen in the cell plasma membrane also affects cell properties like fluidity and signaling during phagocytosis [230].

Peroxisomes are also responsible for cholesterol synthesis. Indeed, several sources state that cholesterol is crucial in signaling and triggering the immune response [234]. Moreover, recent findings suggest that cholesterol synthesis is also crucial for maintaining the integrity and fluidity of the cell membrane and for serving as a precursor for hormone formation. Furthermore, Choi et al., 2021 [235] revealed that cholesterol is essential for autophagy, phagocytosis, and macrophage polarization during inflammation.

**Figure 6:**
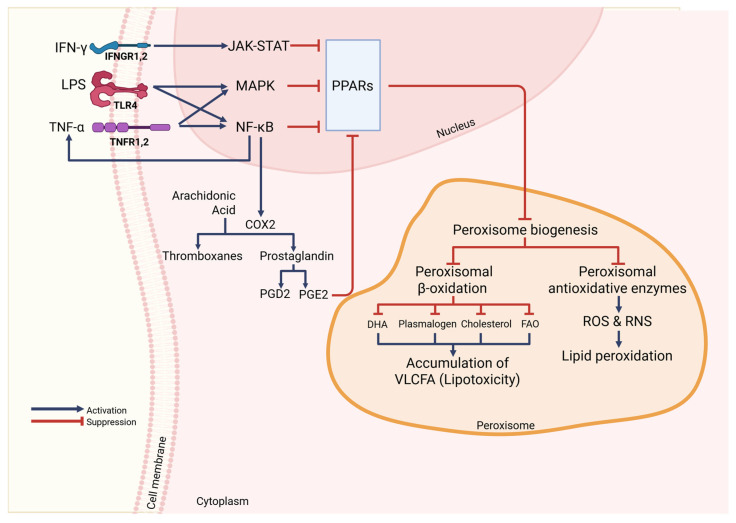
The Role of Peroxisomes and PPARs in Pro-Inflammatory Macrophages. Inflammatory inducers such as LPS, IFN-γ, and TNF-α can negatively affect the functions of peroxisomes and PPARs. LPS signaling induces the TLR4 receptor to inhibit PPAR activity via the NF-κB and MAPK signaling pathways. Activation of NF-κB stimulates the expression of pro-inflammatory cytokines, such as TNF-α, which induces TNFR1 and TNFR2 receptors to reinforce NF-κB and MAPK signaling, thereby further inhibiting PPAR activity. Similarly, IFN-γ induces FNGR1 and FNGR2 receptors to suppress PPAR function through the JAK-STAT signaling pathway. Notably, NF-κB can also downregulate PPARs indirectly by activating COX2, which promotes arachidonic acid metabolism and triggers the production of PGE2, a potent suppressor of PPAR activity. Excessive suppression of PPARs ultimately undermines their ability to activate the transcription of peroxisomal genes. As a result, the essential functions of peroxisomes, such as regulating lipid metabolism and scavenging ROS and RNS, are impaired. This dysfunction leads to lipotoxicity due to the accumulation of VLCFAs and BCFAs that cannot be efficiently metabolized by peroxisomes. Additionally, impaired synthesis of the peroxisomal antioxidant enzyme Catalase results in elevated levels of ROS and RNS, which trigger lipid peroxidation. This series of events contributes to damage to both the cell membrane and intracellular organelles. Abbreviations: IFN-γ (Interferon-γ), IFNGR (Interferon gamma Receptor), JAK-STAT (Janus Kinase-Signal Transducer and Activator of Transcription), LPS (Lipopolysaccharide), TLR4 (Toll-like receptor 4), MAPK (Mitogen-activated protein kinase), TNF-α (Tumor Necrosis Factor-α), TNFR1 (Tumor Necrosis Factor Receptor 1), NF-κB (Nuclear Factor kappa-light-chain-enhancer of activated B cells), PPARs (Peroxisome Proliferator-Activated Receptors), COX2 (Cyclooxygenase-2), PGD2 (Prostaglandin D2), PGE2 (Prostaglandin E2), DHA (Docosahexaenoic acid), FAO (Fatty Acid Oxidation), ROS (Reactive Oxygen Species), RNS (Reactive Nitrogen Species). Created in BioRender. Wihadmadyatami, H. (2025) https://BioRender.com/0molzpx (accessed on 10 December 2025).

Disruptions in the transport and metabolism of fatty acids will undoubtedly substantially impact all these processes. According to Geric et al., 2018 [18], disturbances in any of the peroxisomal β-oxidation enzymes impact DHA production, potentially extending the duration of the inflammatory phase. Raas et al., 2019 [67] found that the absence of *Abcd1*, *Abcd2*, and *Acox1* in mice results in heightened oxidative stress and decreased production of DHA [13]. In particular, the loss of *Mfp2* in the murine peroxisome contributes to inflammation that may develop into more serious issues, such as neuronal dysfunction [18,69,70]. A blockage in the production of plasmalogen and cholesterol will exacerbate the inflammatory condition, as they are crucial for signaling repair and formation of new tissue (Figure 6) [232,236].

### 9.2. Peroxisome Damage Leads to the Accumulation of VLCFA, Inhibits Plasmalogen and Cholesterol Synthesis, and Promotes PPARs

VLCFAs are precursors for lipid mediators that resolve inflammation, and which are sources of energy, and primary components for plasmalogen synthesis and cholesterol production [237]. The peroxisome is the sole organelle able to shorten VLCFA chains before mitochondria can metabolize them [14,26,29]. Notably, peroxisomal dysfunction or damage leads to the accumulation of VLCFA, which triggers lipotoxicity, stemming from the buildup of lipid intermediates in non-adipose tissues, ultimately causing cellular dysfunction and death [238] (Figure 5). Furthermore, impairments in VLCFA catabolism can be detrimental, disrupting lipid balance [230], impacting signaling processes [234], cytotoxicity [239], biosynthesis, apoptosis, autophagy, phagocytosis, and various macrophage functions [235,236] essential for resolving inflammation and polarization [6,7].

The peroxisomal ABCD transporters are the only transporters capable of importing VLCFA as acyl-CoA into the peroxisome [29,37]. Disruptions or damage to peroxisomal *Abcd1* and *Abcd2* can lead to VLCFA accumulation and lipid metabolism imbalance [90]. These interfere with the metabolism of peroxisomal β-oxidation enzymes and impede plasmalogen and cholesterol synthesis [29,90]. Additionally, disruptions in peroxisomal β-oxidation enzymes like *Acox1*, *Mfp2*, and *Acaa1* can also lead to a significant increase in VLCFA levels [18]. Nevertheless, considering their essential function in macrophages, a reduction in the production of plasmalogen and cholesterol will undoubtedly impede the macrophages’ capacity to regulate metabolism and inflammation effectively (Figure 6).

The accumulation of VLCFA triggers the release of the PPARα and PPARγ [17,18,64,240]. This stimulates the upregulation of gene transcription of peroxins, peroxisomal ABCD transporters, peroxisomal β-oxidation enzymes, and plasmalogen synthesis enzymes [18,29,90]. Consequently, there will be a swift rise in peroxisomes, an increase in PPARα and PPARγ, which stimulate the production of anti-inflammatory precursors and encourage macrophage polarization into the M2 phenotype [64,132].

Disruptions in lipid metabolism are not solely caused by damage to peroxisomes, but also dysfunction in other organelles, such as mitochondria and the ER can also impair lipid metabolism. It is well established that peroxisomes do not operate independently in lipid metabolism, rather they work in coordination with other organelles. For instance, in the process of fatty acid oxidation, peroxisomes break down VLCFAs, which can then be further metabolized in mitochondria. Similarly, during the synthesis of plasmalogens and cholesterol, peroxisomes first break down VLCFAs so that these lipids can be produced in the ER. Some of the cholesterol synthesized in the ER is also required in peroxisomes as a precursor for bile acid production [14,17].

### 9.3. Peroxisomes and PPARs Influence Macrophage Polarization, Thereby Activating Phagocytosis and Efferocytosis Phenotypes

Macrophages are cells that play a role in innate immunity and possess the unique capacity to adapt based on the inflammatory environment [5,6]. They transform into the M1 phenotype when stimulated by bacterial infections, such as with LPS or IFN-γ (Figure 6). The M1 phenotype predominantly depends on glycolysis to enhance phagocytosis. In contrast, to resolve inflammation and facilitate tissue repair, macrophages polarize into the M2 phenotype after being induced by IL-4 or IL-13, at which point they predominantly depend on fatty acid oxidation [5,6,206,241,242]. The M1 phenotype produces most of the ROS, which are essential for phagocytosis of intracellular pathogens. This response leads to a metabolic state that favors glycolysis and increases fatty acid production. In contrast, the M2 phenotype generates lower levels of ROS, resulting in a metabolism that relies more on oxidative phosphorylation. This metabolic pathway supports the oxidation of fatty acids, facilitating the phagocytosis of apoptotic cells (efferocytosis), aiding collagen deposition, and playing a critical role in regulating tissue repair [5,18,57,243]. Interestingly, saturated fatty acids can induce macrophages to predominantly polarize into the M1 phenotype, while n-3 PUFA encourages polarization toward the M2 phenotype [80].

Polarization of macrophages has been shown to affect the composition of peroxisomes. In the M1 phenotype, peroxisomal β-oxidation enzymes, such as ACOX1 and MFP2, exhibit a significant decrease. Conversely, in the M2 phenotype, these enzymes increase, which aligns with fatty acid oxidation [18,206]. The enhanced fatty acid metabolism is strongly connected to DHA production, inhibiting inflammation, along with the synthesis of plasmalogen and cholesterol, which aid in tissue repair during inflammation and promote signaling for the release of anti-inflammatory cytokines and the mitigation of oxidative stress [58,64,65,128]. Eventually, peroxisomes have a more prominent role in anti-inflammatory conditions, providing mechanisms to inhibit pro-inflammatory cytokines and promote the repair of damaged tissue.

Numerous studies indicate that PPARγ actively contributes to this polarization process [63,80]. In inflammatory conditions, ROS activates PPARγ, which subsequently initiates the transcription of peroxisomal genes, encourages the polarization of monocytes or macrophages to the M2 phenotype, and enhances fatty acid oxidation [64,66,132]. PPARα and PPARβ also play a role in macrophage reprogramming by regulating fatty acid metabolism through peroxisomal ABCD transporters and peroxisomal β-oxidation enzymes [18,56,206]. PPARβ/δ activation promotes peroxisome proliferation [205,206].

A noteworthy report by Park et al., 2016 [97] identified Catalase as a critical regulator of macrophage polarization in adipose tissue. Decreased Catalase activity heightened inflammation and favored M1 phenotype polarization while suppressing M2 phenotype activation. Furthermore, in macrophages that lacked Catalase, the M1 phenotype was found to be more dominant than the M2 phenotype. These findings underscore the significant role of Catalase in regulating macrophage activation and in maintaining the delicate balance between M1 and M2 phenotypes.

Interestingly, enhancing antioxidant enzyme activity has been suggested to suppress apoptosis. The complete absence of peroxisomes significantly increases apoptosis. Both increased and decreased antioxidant capacity can contribute to carcinogenesis [12,40].

### 9.4. Peroxisomes and PPARs Are Involved in Ferroptosis

Ferroptosis is a form of cell death distinct from apoptosis. It typically occurs due to the accumulation of iron and a deficiency in the antioxidant enzymes glutathione (GSH) and glutathione peroxidase 4 (GPX4). This imbalance leads to lipid peroxidation, particularly PUFAs, resulting in the uncontrolled production of ROS [221,244]. Macrophages are the immune cells that play the most critical role during ferroptosis. They are responsible for maintaining tissue homeostasis by regulating inflammation, as well as iron, lipid, and amino acid metabolism. In the context of ferroptosis regulation, macrophages perform several functions, including reprogramming through the secretion of pro- or anti-inflammatory cytokines, as well as engaging in phagocytosis and efferocytosis [230].

Peroxisomes and PPARs contribute to ferroptosis. They are actively involved in both the initiation and resolution of this process. The synthesis of PUFA-plasmalogens and arachidonic acid/adrenic acid-phosphatidylethanolamine (AA/AdA-PE) occurs through both peroxisome-dependent and independent pathways. The production of lipid hydroperoxide (LOOH) from these lipids is a key step in promoting ferroptosis [244]. FAR1-mediated ferroptosis depends on the biosynthesis of ether phospholipids, a process facilitated by peroxisomes. When FAR1 is inactivated, saturated fatty acid-dependent ferroptosis is significantly reduced [245].

Interestingly, PEX3 and PEX10 have been identified to play a role in the resolution of ferroptosis [222]. Conversely, deficiency of PEX3 and PEX10 reduces cellular sensitivity to ferroptosis induced by GPX4 inhibition. PPARβ/δ has been shown to inhibit ferroptosis in murine embryonic fibroblasts, leading to increased expression of Catalase [223]. Additionally, PPARγ reduces neuronal ferroptosis by downregulating COX2 [224].

Although there is no specific information yet regarding the role of peroxisomes in macrophages during ferroptosis, existing literature provides sufficient insight to illustrate how peroxisomes may function in macrophages throughout the ferroptosis process. Peroxisomes contribute to the resolution of ferroptosis by synthesizing plasmalogens, which help replace damaged membrane lipids resulting from lipid peroxidation. Ferroptosis can be exacerbated when plasmalogen synthesis by peroxisomes is impaired. The accumulation of ROS caused by lipid peroxidation can be neutralized through increased production of peroxisomal antioxidant enzymes. PPARs play a key role in regulating the transcription of peroxisomal genes, and their activation can be triggered by elevated levels of ROS and lipid peroxidation, as well as a number of macrophages, to accelerate the resolution of ferroptosis.

## 10. Translational Perspective: Targeting PPARs in Macrophage-Mediated Diseases

### 10.1. Clinically Approved PPARs Agonist

PPARs play a vital role in regulating genes associated with lipid metabolism, glucose homeostasis, inflammation, and immune responses in macrophages. They also influence macrophage polarization and function. Targeting PPARs in macrophages may provide therapeutic benefits for chronic inflammatory and metabolic diseases, including atherosclerosis, type 2 diabetes, obesity, and non-alcoholic fatty liver disease (NAFLD) [49,56,204,212]. Currently, only PPARα and PPARγ agonists are well-established and utilized in treating various diseases that target PPARs in macrophages. Meanwhile, PPARs agonists have also been investigated. Especially, the use of PPAR-β/δ agonists remains predominantly confined to experimental studies [246,247,248].

PPAR-α agonists, commonly known as fibrates, including fenofibrate, gemfibrozil, and bezafibrate, are used to treat hyperlipidemia and hypertriglyceridemia. In macrophages, these drugs suppress the production of pro-inflammatory cytokines, such as TNF-α and IL-6. They also promote fatty acid oxidation, reduce the formation of foam cells which helps counteract atherosclerosis, and shift macrophage polarization from the M1 to the M2 phenotype, aiding in the resolution of inflammation [57,60,204].

PPAR-γ agonists, particularly thiazolidinediones (TZDs) such as rosiglitazone and pioglitazone, have been widely used in the treatment of type 2 diabetes mellitus. They exert their effects by promoting the M2 phenotype, thereby enhancing the resolution of inflammation. Additionally, they inhibit NF-κB signaling, leading to reduced expression of IL-1β, TNF-α, and iNOS. These agonists also improve insulin sensitivity by modulating macrophage-driven inflammation in adipose tissue and support efferocytosis, the clearance of apoptotic cells, in atherosclerotic lesions [56,57,60,212,249]. However, despite their therapeutic efficacy, safety concerns such as cardiovascular risks and fluid retention have limited the broader clinical use of certain PPAR agonists, particularly those targeting PPAR-γ [246,247,248]. Interestingly, the pioglitazone derivative leriglitazone [250] has shown clinical benefits in alleviating symptoms in patients with neuroinflammatory and neurodegenerative disorders, including X-ALD and Friedreich’s ataxia [251,252].

Currently, PPAR-β/δ agonists, such as GW501516, remain unapproved due to safety concerns, particularly regarding their potential carcinogenicity. As a result, their use is primarily restricted to experimental research. In these studies, the potential benefits of these agonists have been investigated, including their ability to enhance fatty acid oxidation, promote the M2 phenotype, improve metabolic reprogramming, and potentially reduce inflammation in models of atherosclerosis and obesity [60,246,247,248].

Targeting PPARs in macrophages shows great potential for treating various metabolic and inflammatory diseases. Although current agonists, such as fibrates and thiazolidinediones (TZDs), have proven benefits, new strategies that emphasize selectivity, safety, and targeted action in macrophages are expected to influence the future of immunometabolic therapies [56,60,248].

### 10.2. Emerging Therapeutic Strategies

Various new strategies are being researched to modulate PPAR signaling in macrophages with improved specificity and safety. These include dual or pan-PPAR agonists [253,254], selective PPAR modulators (SPPARMs) [255], natural ligands and dietary components [60], and targeted drug delivery [256].

The dual or pan-PPAR agonist approach involves stimulating two or more PPAR isoforms simultaneously using compounds such as elafibranor, a dual PPAR-α/δ agonist [253,254] and PPARα/γ dual agonist tesaglitazar [257]. This strategy has shown enhanced effectiveness in managing complex diseases like non-alcoholic steatohepatitis (NASH) by modulating various metabolic pathways and influencing macrophage functions [258].

The SPPARMs model employs agents designed to retain the therapeutic benefits of full agonists while reducing unwanted side effects. For instance, INT131 is a selective PPAR-γ modulator that has demonstrated fewer adverse effects in the treatment of diabetes [259,260].

The natural ligands and dietary components pathway involve the use of endogenous ligands, such as fatty acids, eicosanoids, and phytochemicals (e.g., resveratrol and omega-3 fatty acids). These compounds have demonstrated the potential to activate PPARs in macrophages while posing fewer safety concerns [60,261].

In targeted drug delivery, PPARs can be integrated into innovative delivery systems, such as nanoparticles [262], nanospheres [263], and liposomes [257]. These advanced systems are being designed to enhance the specificity of PPAR agonists for macrophages in diseased tissues, thereby minimizing systemic toxicity [257,262,263].

### 10.3. Future Directions

Emerging research reveals that PPARs in macrophages play more diverse and critical roles than previously recognized, opening promising avenues for future therapeutic strategies. Precision medicine approaches, such as single-cell sequencing and lipidomics, hold potential for identifying disease-specific macrophage subtypes, paving the way for tailored PPAR-targeted treatments. In parallel, macrophage-specific modulation using gene therapy or RNA-based technologies may allow for selective activation or silencing of PPARs exclusively within macrophages, minimizing off-target effects [248,256,264].

Moreover, combining PPAR modulators with other therapeutic agents, such as anti-inflammatory biologics, immune checkpoint inhibitors, or metabolic drugs, could enhance treatment efficacy for complex diseases like atherosclerosis, diabetes, cancer, and autoimmune disorders. Additionally, expanding investigations into lesser-known roles of PPARs, including their involvement in efferocytosis, angiogenesis, and tissue repair, may further broaden their clinical applications and reinforce their significance in immune and metabolic regulation [248,256].

## 11. Conclusions

This review highlights the pivotal roles of peroxisomes and PPARs in coordinating macrophage metabolism, redox regulation, and immune function. By modulating lipid metabolism, ROS and RNS detoxification, and gene transcription, the peroxisome–PPAR axis drives macrophage polarization and inflammatory resolution. These insights offer potential therapeutic avenues in treating chronic inflammatory, metabolic, and degenerative diseases.

## Figures and Tables

**Table 1:** Organ-Resident Macrophages, Peroxisomes, PPARs, and Their Functions.

Resident Macrophages	Known PO Markers	Presence of PPARs	Functions of Peroxisomes and PPARs	Preventing Diseases	Ref.
Brain and CNS					
Microglia, BAMs (perivascular, meningeal, and choroid plexus macrophages)	ABCD1, ABCD2, ABCD3, ACOX1, MFP2, PEX1, PEX5L, PEX11β, MFP2, and CAT	PPARα, PPARβ/δ, and PPARγ	Maintaining nervous system homeostasis, suppressing inflammation, repairing tissue damage, phagocytosis of myelin debris, triggering the recruitment of oligodendrocyte precursor cells, and apoptotic cells	Cancer, neurodegenerative disorders (X-ALD), multiple sclerosis, neuronal dysfunction, and behavioral abnormalities	[7,8,67,68,69,70,71,72]
Lungs					
Alveolar macrophages and Interstitial macrophages	PEX5, PEX14, ABCD3, ACOX1, ACAA1, CAT and GPX	PPARγ	Maintaining pulmonary organ homeostasis, balancing defense against pathogens, pollutants, allergens, and toxins, as well as tolerance towards harmless stimuli	COPD, pulmonary alveolar proteinosis, cystic fibrosis, influenza, asthma, and allergic reactions	[9,10,73,74]
Liver					
Kupffer cells, monocyte-derived liver macrophages, and capsular macrophages	CAT	PPARα and PPARγ	Maintaining liver homeostasis, scavenging bacterial infections, contributing to the development of both acute and chronic liver injuries, monitoring the gut–liver axis for pathogens and toxins, clearing cellular debris and metabolites, aiding liver tissue repair, and helping maintain iron balance	Alcohol-associated liver disease, hepatic steatosis, alcoholic and non-alcoholic steatohepatitis, liver cancer, autoimmune hepatitis, toxic liver injury, and viral hepatitis	[75,76,77,78,79,80,81]
Spleen					
Red pulp macrophages, white pulp macrophages, marginal zone macrophages, and marginal metallophilic macrophages	-	PPARγ	Maintaining blood-borne infections, scavengers for senescent erythrocytes, oxidizing iron, assisting in controlling infections, and internalizing pathogens present in the bloodstream	Bacterial, viral, and yeast infections	[82]
Kidneys					
Renal macrophages (renal medulla macrophages or juxtatubular macrophages, glomerular macrophages, and interstitial macrophages)	-	PPARα and PPARγ	Monitor and clear urine particles to prevent tubular obstruction, assist in detoxifying uremic toxins,	Renal fibrosis, diabetic kidney disease, chronic kidney disease, cancers, accelerated hyperoxaluria, and kidney stones	[83,84,85]
Heart					
Cardiac macrophages	-	PPARα, PPARβ/δ, and PPARγ	Maintaining cardiovascular system balance, regulating inflammation, facilitating cardiac remodeling, clearing cellular debris, modulating arterial tone, patrolling blood vessels, remodeling heart valves, supporting osmoregulation, and contributing to electrical conduction	Cardiac fibrosis, myocardial infarction, arrhythmia, obesity, hypertension, diabetes, myocarditis, and ischemia	[86,87]
Intestine					
Intestinal macrophages (mucosal macrophages, lamina propria macrophages, muscularis macrophages, perivascular macrophages, ileal macrophages, and colonic macrophages	-	-	Preventing infections from pathogens entering the gastrointestinal tract, maintaining immune homeostasis, regulating inflammatory responses, repairing damaged tissues, clearing dead cells and foreign debris, and controlling gut motility and secretion	Postoperative ileus, inflammatory bowel disease, necrotizing enterocolitis, and gastrointestinal disorders associated with HIV/AIDS and Parkinson’s disease	[88,89]
Peritoneal Cavity					
Peritoneal macrophages	PEX14, ABCD1, ABCD2, MFP2, and ACAA1	PPARα and PPARγ	Regulate peritoneal B1 cells and migrate via nonvascular pathways to the neighboring organs, inhibit T cell proliferation, and mitigate inflammation in neighboring tissues	Sepsis, cancers, endometriosis, ascites, and peritoneal injuries leading to abdominal surgery	[10,18,74,90,91,92,93]
Bone and joints					
Osteoclasts and synovial macrophages	PEX14	PPARγ	Maintaining bone resorption, bone remodeling, and inflammatory processes, facilitates the dissolution of minerals and the degradation of the bone matrix, secretes regulatory factors contributing to cartilage and bone turnover, while clearing cellular debris and pathogens to prevent sterile and septic inflammation	Rheumatoid arthritis, osteoporosis, periprosthetic osteolysis, osteolytic lesions in myeloma, and periodontitis	[94,95,96]
Adipose tissue					
Adipose tissue macrophages	PEX14, ACAA1, and CAT	PPARγ	Regulating lipid metabolism and inflammation in obesity	Adipocyte dysfunction, fibrosis associated with obesity, and obesity-induced insulin resistance	[74,97,98,99]
Reproductive Organs					
Testicular macrophages (peritubular macrophages and interstitial testicular macrophages), ovarian macrophages, and uterine macrophages	-	PPARα and PPARγ	Maintaining reproductive organs’ homeostasis (lipid metabolism, inflammation, hormones), barrier against bacterial infections, regulating fertility, and embryo implantation	Bacterial and viral infections in reproductive organs	[100,101,102]
Lymph Nodes					
Subcapsular sinus macrophages, medullary sinus macrophages, medullary cord macrophages, and interfollicular macrophages	-	-	Trapping and presenting antigens to B cells, preventing the systemic dissemination of pathogens carried through the lymph, and lymphatic immune defense against a range of infections	Cancer, colitis, bacterial, and viral infections	[103,104,105,106]
Pancreas					
Islet macrophages	-	PPARα and PPARγ	Regulating pancreatic vascular remodeling, islet structure remodeling, insulin secretion capacity, glucose homeostasis, lipid metabolism, inflammation control, and the production of connective tissue growth factors	Obesity and diabetes	[107,108,109]
Skin					
Langerhans cells and dermal macrophages	-	PPARγ	The first line of defense against invading pathogens and is crucial for controlling inflammation, promoting wound healing, regulating lipid metabolism, and maintaining ROS balance in the skin	Dermatological disorders, psoriasis, chronic cutaneous diseases, dermatopathic lymphadenopathy, and neoplasms	[110,111,112]
Eyes					
Vitreal macrophages (hyalocytes), microglia, perivascular macrophages, and monocyte-derived macrophages	-	PPARα	Maintaining the balance of inflammation, immune cell migration, and performing erythrophagocytosis, essential for ocular neurovascularization, and vascular development	Diabetic retinopathy, uveitis, retinal ischemia, and neurotoxic retinal degeneration	[113,114]
Skeletal Muscle					
Muscle macrophages	-	PPARγ	Promotes skeletal muscle regeneration, helps modulate the inflammatory response during injury or metabolic stress, favoring a pro-repair phenotype	Muscular dystrophy, including the differentiation of satellite cells and abnormal muscle regeneration, contributes to chronic muscle diseases	[115,116,117]

Abbreviations: PO (peroxisomes), Ref. (References), BAMs (border-associated macrophages), ABCD (ATP-binding cassette transporter subfamily D), ACOX (Acyl-coenzyme A oxidase), PEX (Peroxin), MFP (MultiFunctional Protein), CAT (Catalase), PPAR (Peroxisome Proliferator-Activated Receptor), GPX (Glutathione Peroxidase), ACAA1 (3-ketoacyl-CoA thiolase), X-ALD (X-linked adrenoleukodystrophy), COPD (chronic obstructive pulmonary disease), HIV/AIDS (human immunodeficiency virus/Acquired Immune Deficiency Syndrome).

**Table 2:** Presence of Peroxisomal Proteins, PPARs, and Their Functional Relevance in Primary/Immortal Macrophages.

Species	Known PO Markers	Presence of PPARs	Functions of Peroxisomes and PPARs	Preventing Diseases	Ref.
Bone Marrow-derived Macrophages (BMDMs)					
Human, murine	PEX5, PEX11β, PEX13, PEX14, PEX19, ACOX1, MFP2, ACAA1, CAT, and ABCD3	PPARα and PPARγ	Play an essential role in the formation of tissue-resident macrophages in skin, intestine, lymph, and heart tissue	Atherosclerosis disease	[2,15,132,202,203,204,205,206,207]
RAW264.7 Cell Line					
Murine	PEX5, PEX14, ABCD3, and MFP2	PPARγ	Regulating lipid metabolism and inflammation	-	[10,15,174,203,208,209]
Induced pluripotent stem cell (iPSC)-derived human macrophages (iPSDMs)					
Human	PEX3, PEX14, ABCD3, ACOX1, HSD, and CAT	PPARα, PPARβ/δ, and PPARγ	Control of cytosolic and ROS	Mycobacterium tuberculosis infection	[210]
Human peripheral blood mononuclear-derived macrophages					
Human	-	PPARα and PPARγ	Enhancing efferocytosis in the M2 phenotype	Atherosclerosis	[56,66,92,211]
Embryonic stem-derived macrophages					
Human	-	PPARα and PPARγ	Regulating lipid metabolism and inflammation	-	[212]
Tumor-associated macrophages (TAMs)					
Murine	-	PPARα, PPARβ/δ, and PPARγ	Regulating their phenotype, inhibiting the angiogenesis of the tumor, and promoting immunostimulatory activities	Tumors and cancers	[93]

Abbreviations: PO (peroxisomes), Ref. (References), PEX (Peroxin), ACOX1 (Acyl-coenzyme A oxidase 1), MFP2 (MultiFunctional Protein 2), ACAA1 (3-ketoacyl-CoA thiolase), CAT (Catalase), ABCD (ATP-binding cassette transporter subfamily D), PPAR (Peroxisome Proliferator-Activated Receptor), HSD (Hydroxysteroid Dehydrogenase).

**Table 3:** The Role of Peroxisomes and PPARs in Supporting Macrophage Function.

Macrophage Function	Role of Peroxisomes	Role of PPARs	Ref.
Regulation of Inflammation (defense against pathogens, clearance of dead cells and debris, and wound healing and tissue repair	Regulation of FAO, plasmalogen, DHA, and cholesterol synthesis	Regulating peroxisomal genes to maintain inflammatory balance	[10,13,17]
Scavenge ROS and RNS	Producing peroxisomal antioxidative enzymes and synthesizing plasmalogens	Maintaining the balance of peroxisomal antioxidative enzyme production and plasmalogen synthesis	[10,13]
Ferroptosis	Regulating peroxisomal antioxidative enzymes for ROS and RNS balance. Lipid homeostasis.	Initiation and resolution by macrophage reprogramming	[221,222,223,224]
Angiogenesis	Involvement in lipid metabolism, redox balance	Regulating phenotype, inhibiting the angiogenesis of the tumor, and promoting immunostimulatory activities	[61,93]
Metabolic and Homeostasis Functions	Lipid metabolism, peroxisomal antioxidative enzymes regulation	Regulating peroxisomal genes	[10,18]

Abbreviations: Ref. (References), PPARs (Peroxisome Proliferator-Activated Receptors), FAO (Fatty Acid Oxidation), DHA (Docosahexaenoic acid), ROS (Reactive Oxygen Species), RNS (Reactive Nitrogen Species).

## Data Availability

No new data were created or analyzed in this study.
